# Synthesis and the Biological Activity of Phosphonylated 1,2,3-Triazolenaphthalimide Conjugates

**DOI:** 10.3390/molecules21111420

**Published:** 2016-10-26

**Authors:** Iwona E. Głowacka, Rafał Gulej, Piotr Grzonkowski, Graciela Andrei, Dominique Schols, Robert Snoeck, Dorota G. Piotrowska

**Affiliations:** 1 Bioorganic Chemistry Laboratory, Faculty of Pharmacy, Medical University of Lodz, Muszyńskiego 1, 90-151 Lodz, Poland; rafal.gulej@stud.umed.lodz.pl (R.G.); grzonkowskipiotr4@gmail.com (P.G.); dorota.piotrowska@umed.lodz.pl (D.G.P.); 2 Rega Institute for Medical Research, KU Leuven, Minderbroedersstraat 10, B-3000 Leuven, Belgium; Graciela.Andrei@rega.kleuven.be (G.A.); Dominique.Schols@rega.kleuven.be (D.S.); Robert.Snoeck@rega.kleuven.be (R.S.)

**Keywords:** azidophosphonates, acyclonucleotides, naphthalimides, 1,2,3-triazoles, cycloaddition, antiviral, cytostatic

## Abstract

A novel series of diethyl {4-[(5-substituted-1,3-dioxo-1*H*-benzo[*de*]isoquinolin-2(3*H*)-yl)-methyl]-1*H*-1,2,3-triazol-1-yl}alkylphosphonates designed as analogues of amonafide was synthesized. All phosphonates were assessed for antiviral activity against a broad range of DNA and RNA viruses and several of them showed potency against varicella-zoster virus (VZV) [EC_50_ (50% effective concentration) = 27.6–91.5 μM]. Compound **16b** exhibited the highest activity against a thymidine kinase-deficient (TK^−^) VZV strain (EC_50_ = 27.59 μM), while **16d** was the most potent towards TK^+^ VZV (EC_50_ = 29.91 μM). Cytostatic properties of the compounds **14a**–**i**–**17a**–**i** were studied on L1210, CEM, HeLa and HMEC-1 cell lines and most of them were slightly cytostatic for HeLa [IC_50_ (50% inhibitory concentration) = 29–130 µM] and L1210 cells [IC_50_ (50% inhibitory concentration) = 14–142 µM].

## 1. Introduction

Pharmacologically important natural as well as synthetic compounds having various heterocyclic systems including powerful pharmacophores such as triazoles and naphthalimides are of special interest. Numerous compounds of the 1,2,3-triazole family have shown a broad spectrum of biological activities, including antibacterial [1,2], antifungal [3,4,5,6], anticancer [7,8,9,10,11], antiviral [12,13,14,15,16] and antiallergic effects [17]. Furthermore, substituted 1,2,3-triazoles have also been used as agrochemicals, dyes, photostabilisers and corrosion inhibitors [18,19,20]. Various biological activities of substituted 1,2,3-triazoles are closely related to their chemical reactivity since they are able to form hydrogen bonds, could be protonated at the physiological pH and are resistant to oxidation and reduction as well as to enzymatic hydrolysis to name the most important features.

Naphthalimide derivatives such as mitonafide [21,22,23], amonafide [21,22,23,24,25], azonafide [26,27,28], DMP-840 [29] and elinafide (Lu-79553) [30,31] exhibit intercalating properties [21,22]. However, the clinical use of these compounds has been limited due to their low therapeutic indices as well as poor water-solubility [32]. In order to improve therapeutic properties of naphthalimides, many efforts have been undertaken towards synthesizing novel derivatives with higher activity and lower toxicity.

**Figure 1:**
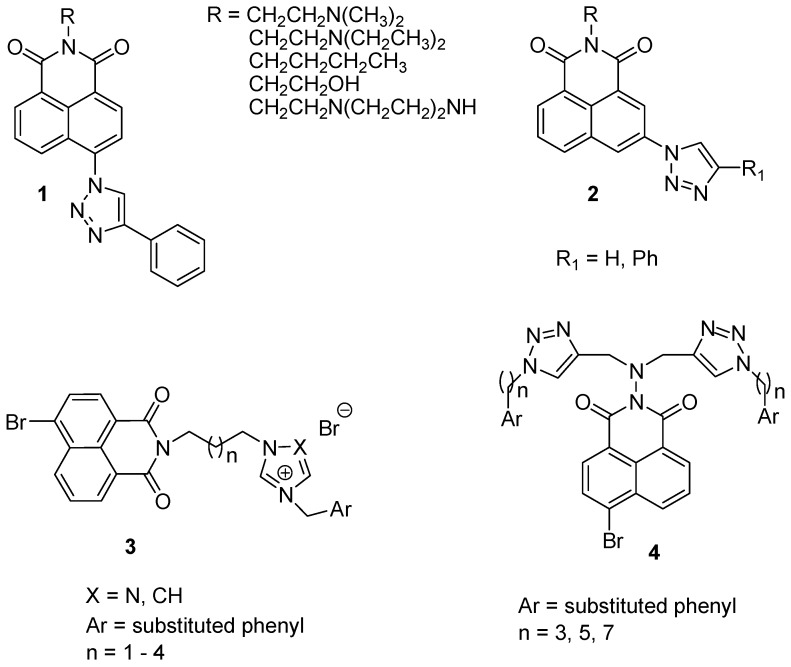
Examples of naphthalimides containing triazole units.

The concept of combining two pharmacophoric fragments of biologically active compounds into a single molecule is commonly applied aiming at improvement of activities and eventually to avoid serious side effects of the known candidates. Having this idea in mind several structural analogues of amonafide have been synthesized over the years (Figure 1).

Qian and Li reported the synthesis of 6-(1,2,3-triazole)-1,8-naphthalimides **1** and proved their cytotoxic activity [33]. Furthermore, 5-substituted analogues **2** were also obtained and their cytotoxicity against MCF-7, HeLa and 7721 cells was evaluated [34]. Among all tested compounds **2**, derivatives having a 2-(*N*,*N*-dimethylamino)ethyl group at the imide nitrogen and phenyl at C4 in the 1,2,3-triazole ring or alternatively lacking this substituent were found the most active (IC_50_ in the 0.258–0.725 μM range) with inhibition abilities higher than that of amonafide, used as a control. On the other hand, antifungal and antimicrobial properties of 1,2,4-triazole derivatives **3** were examined and several compounds exhibited even better activity against some tested strains than orbifloxacin, chloromycin and fluconazole used as reference drugs [35]. Among bis(1,2,3-triazole)-conjugates of naphthalimides **4**, a derivative substituted with 3,4-dichlorophenyl groups exhibited better inhibitory activity toward *Escherichia coli* than norfloxacin and chloromycin with a minimum inhibitory concentration (MIC) value of 1 μg/mL [36].

These achievements prompted us to propose a new modification at the imide nitrogen of amonafide **14**–**17** by installation of N1-substituted 1,2,3-triazoles decorated at the end of the alkyl chain with phosphonoalkyl groups. We aimed to understand the influence of the phosphonate group on the biological activity of the designed amonafide analogues. On the other hand, compounds **14**–**17** resemble analogues of acyclic nucleotides in which the phosphate group is replaced with a phosphonate moiety and a naphthalimide fragment serves as a modified nucleobase. Thus, in principle compounds **14**–**17** may primarily act as intercalators (through a naphthalimide ring) but also by a phosphonate activation and termination of DNA/RNA synthesis.

## 2. Results and Discussion

### 2.1. Chemistry

**Scheme 1:**
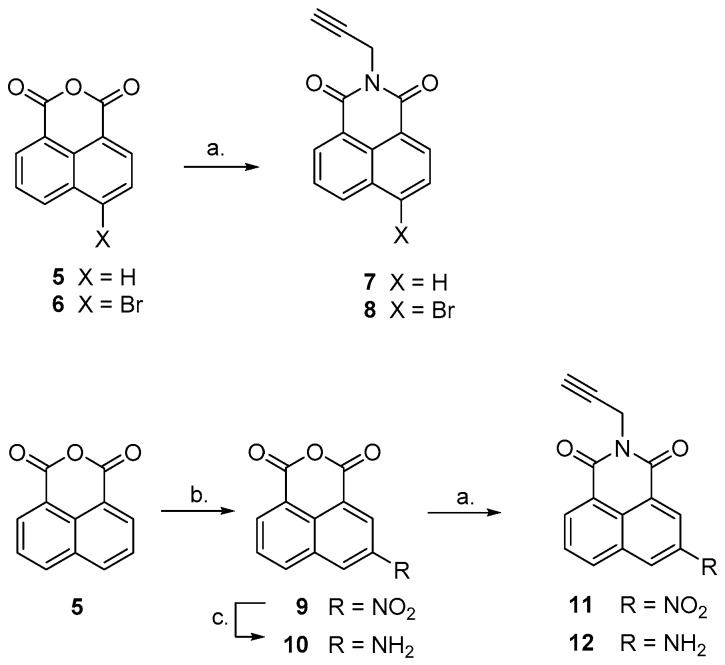
Synthesis of the alkynes **7**, **8**, **11** and **12**. *Reagents and Conditions*: a. propargylamine, EtOH, reflux (3 h for **7**, **11** and **12**; 20 h for **8**); b. HNO_3_, H_2_SO_4_; c. SnCl_2_, HCl.

**Scheme 2:**
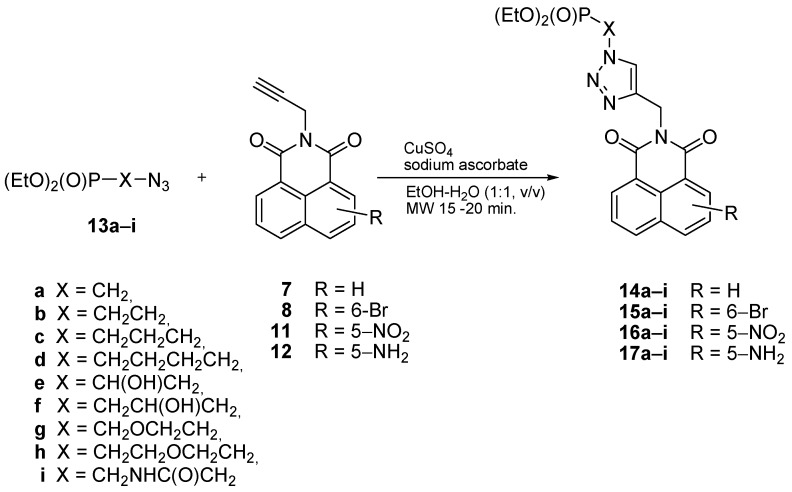
Synthesis of 1,2,3-triazoles derivatives **14a–i**–**17a**–**i**.

The synthetic strategy to the 1,2,3-triazole-containing naphthalimide derivatives **14a**–**i**–**17a**–**i** is presented in Scheme 1 and Scheme 2. The syntheses of alkynes **7** [37,38,39] and **8** [40,41,42] have been previously described. For the purpose of this project both **7** and **8** were obtained following the known procedure described for **8** [41], although it appeared to be a new approach to the preparation of a compound **7**. The alkynes **11** and **12** were synthesized from commercially available 1,8-naphthalimic anhydride **5** as shown in Scheme 1. The nitration of the anhydride **5** provided **9** [43] which was then reduced to give a compound **10** [44]. The subsequent propargylation of **9** and **10** afforded the alkynes **11** and **12**, respectively. Azidophosphonates **13a**–**i** [45,46,47,48,49,50] were previously obtained and fully characterized in our laboratory. The Cu(I)-catalyzed Hüisgen dipolar cycloaddition of *N*-propargyl derivatives **7**–**8**/**11**–**12** and the respective azidoalkylphosphonates **13a**–**i** [45,46,47,48,49,50] allowed to construct naphthalimides **14a**–**i**–**17a**–**i** having various phosphonoalkyl groups regioselectively installed at N-1 of the 1,2,3-triazole subunit (Scheme 2). The chromatographic purification or crystallization gave pure **14a**–**i**–**17a**–**i** in good to excellent yields. Their structures and purities were established by ^1^H, ^13^C and ^31^P NMR and IR techniques as well as by elemental analyses.

### 2.2. Antiviral Activity and Cytostatic/Cytotoxic Evaluation

Phosphonates **14a**–**i**–**17a**–**i** were evaluated for their antiviral activities against a wide variety of DNA and RNA viruses using the following cell-based assays: (a) human embryonic lung (HEL) cell cultures: herpes simplex virus-1 (KOS strain), herpes simplex virus-2 (G strain), vaccinia virus, vesicular stomatitis virus, thymidine kinase-deficient herpes simplex virus-1 (TK^−^ KOS ACV^r^) and adenovirus-2, cytomegalovirus (AD-169 stain and Davis stains) and varicella-zoster virus (TK+ VZV stain and TK^−^ VZV stain); (b) HeLa cell cultures: vesicular stomatitis virus, Coxsackie virus B4 and respiratory syncytial virus; (c) Vero cell cultures: para-influenza-3 virus, reovirus-1, Sindbis virus, Coxsackie virus B4, Punta Toro virus; (d) Crandell-Rees Feline Kidney (CRFK) cell cultures: feline corona virus (FIPV) and feline herpes virus (FHV) and (e) Madin Darby Canine Kidney (MDCK) cell cultures: influenza A virus H1N1 subtype, influenza A virus H3N2 subtype and influenza B virus. Ganciclovir, cidofovir, acyclovir, brivudin, (*S*)-9-(2,3-dihydroxypropyl)adenine [(*S*)-DHPA], *Hippeastrum* hybrid agglutinin (HHA), *Urticadioica* agglutinin (UDA), dextran sulfate (molecular weight 5000, DS-5000), ribavirin, oseltamivir carboxylate, amantadine and rimantadine were used as the reference compounds. The antiviral activity was expressed as the EC_50_: the compound concentration required to reduce virus-induced cytopathogenicity by 50% (other viruses).

Among the synthesized compounds, several phosphonates **14**, **15** and **16** slightly inhibited the replication of both TK^+^ and TK^−^ VZV strains with EC_50_ in the 27.6–91.5 μM range, however with lower potency than that of acyclovir and brivudine, used as reference drugs (Table 1).

The cytotoxicity of the tested compounds toward the uninfected host cells was defined as the minimum cytotoxic concentration (MCC) that causes a microscopically detectable alteration of normal cell morphology. The 50% cytotoxic concentration (CC_50_), causing a 50% decrease in cell viability was determined using a colorimetric 3-(4,5-dimethylthiazol-2-yl)-5-(3-carboxymethoxy-phenyl)-2-(4-sulfophenyl)-2*H*-tetrazolium (MTS) assay system. None of the tested compounds affected cell morphology of HEL, HeLa, Vero, MDCK and CRFK cells at concentrations up to 100 μM.

The cytostatic activity of the tested compounds was defined as the 50% cytostatic inhibitory concentration (IC_50_) causing a 50% decrease in cell proliferation and was determined against murine leukaemia L1210, human lymphocyte CEM, human cervix carcinoma HeLa and human dermal microvascular endothelial HMEC-1 cells (Table 2).

Among all tested compounds 1,2,3-triazole-amonafide conjugates **15a**–**i** having a bromine atom at C6 of the naphthalimide unit were the most cystostatic toward the tested tumor cell lines at concentrations as low as 14 μM being especially effective for L1210 (IC_50_ = 14–42 μM). Conjugates **16a**–**i** containing the nitro group at C5 were slightly less active and showed moderate cytostatic effects toward HeLa cells (IC_50_ = 29–132 μM). The replacement of the nitro by an amino group at C5 of the naphthalimide skeleton resulted in the decrease or even loss of the inhibitory capacity of the respective analogues (**15a**–**i** vs. **17a**–**i**). Similarly, negligible inhibitory properties against the proliferation of the tested cell lines were noticed for the series of naphthalimide phosphonates **14a**–**i** (R = H).

The presence of the 1,2,3-triazole unit seems to be necessary for cytostatic activity of the tested compounds since naphthalimides devoid of this moiety appeared inactive (**11** vs. **16a**–**i** and **8** vs. **15a**–**i**). However, it was found that a compound **7** moderately inhibited (IC_50_ = 48 μM) the proliferation of HeLa cells while naphthalimides **14a**–**i** could be considered inactive (IC_50_ = 150–>250 μM). Among the 1,2,3-triazoles with phosphonate linkers [compounds **16** (nitro) and **15** (bromo)], the compounds with longer fragments are generally associated with the higher potency, e.g., **16c** and **15c** (trimethylene), **15d** (tetramethylene) and **15h** (CH_2_CH_2_OCH_2_CH_2_), the lowest activity being observed for **16e** and **15e** [CH(OH)CH_2_] as well as for **16i** and **15i** [CH_2_NHC(O)CH_2_] with shorter fragments.

## 3. Experimental Section

### 3.1. General Information

^1^H-NMR spectra were taken in CDCl_3_ or DMSO-*d*_6_ on the following spectrometers: Mercury-200 (Varian NMR INSTRUMENT, Palo Alto, CA, USA) and Avance III (600 MHz) (Bruker Instruments, Karlsruhe, Germany) with TMS as an internal standard; chemical shifts δ are given in ppm with respect to TMS and coupling constants *J* in Hz. ^13^C-NMR spectra were recorded for CDCl_3_ or DMSO-*d*_6_ solutions on a Bruker Avance III (600 MHz) spectrometer at 151 MHz. ^31^P-NMR spectra were taken in CDCl_3_ or DMSO-*d*_6_ on Varian Mercury-200 and Bruker Avance III at 81 and 243 MHz.

IR spectral data were measured on an Infinity MI-60 FT-IR spectrometer (ATI Instruments North America—Mattson, Medison, WI, USA). Melting points were determined on a Boetius apparatus and are uncorrected. Elemental analyses were performed by the Microanalytical Laboratory of the Faculty of Pharmacy (Medical University of Lodz) on a PE 2400 CHNS analyser (Perkin Elmer Corp., Norwalk, CT, USA).

The following adsorbents were used: column chromatography, silica gel 60 (70–230 mesh, Merck KGaA, Darmstadt, Germany); analytical TLC, Merck TLC plastic sheets silica gel 60 F_254_. TLC plates were developed in chloroform–methanol solvent systems. Visualization of spots was effected with iodine vapors. All solvents were purified by methods described in the literature.

All microwave irradiation experiments were carried out in a RM 800 microwave reactor (Plazmatronika, Wrocław, Poland).

### 3.2. Synthesis of 2-(Prop-2-yn-1-yl)-1H-benzo[de]isoquinoline-1,3(2H)-dione *(**7**)*

A suspension of a compound **5** (1.00 mmol), propargyl amine (1.05 mmol) in ethanol (15 mL) was stirred at 78 °C for 3 h. The reaction mixture was cooled to room temperature and filtered to give pure **7** as a white powder. Yield 90%; m.p. 239–240 °C; IR (KBr): ν = 3244, 3001, 2996, 1734, 1689, 1331, 780 cm^−1^; ^1^H-NMR (200 MHz, CDCl_3_): δ = 8.65 (dd, *J* = 7.3 Hz, *J* = 1.2 Hz, 2H, H_aromat._), 8.24 (dd, *J* = 8.1 Hz, *J* = 1.2 Hz, 2H, H_aromat._), 7.78 (dd, *J* = 8.1 Hz, *J* = 7.3 Hz, 2H, H_aromat._), 4.97 (d, *J* = 2.5 Hz, 1H, *H*C≡CCH_2_), 2.20 (t, *J* = 2.5 Hz, 2H, HC≡CC*H*_2_).

### 3.3. Synthesis of 5-Nitro-2-(prop-2-yn-1-yl)-1H-benzo[de]isoquinoline-1,3(2H)-dione *(**11**)*

A suspension of compound **9** (1.00 mmol), propargyl amine (1.05 mmol) in ethanol (15 mL) was stirred at 78 °C for 4 h. The reaction mixture was cooled to room temperature then filtered to give **11** as an orange powder which was pure to be used in the next step without further purification. Yield 90%; m.p. = 213–214 °C; IR (KBr): ν = 3257, 3082, 3064, 2992, 1711, 1672, 1344, 789 cm^−1^; ^1^H-NMR (200 MHz, CDCl_3_): δ = 9.30 (d, *J* = 2.2 Hz, 1H, H_aromat._), 9.11 (d, *J* = 2.2 Hz, 1H, H_aromat._), 8.78 (dd, *J* = 7.3 Hz, *J* = 1.2 Hz, 1H, H_aromat._), 8.40 (dd, *J* = 8.3 Hz, *J* = 1.2 Hz, 1H, H_aromat._), 7.91 (dd, *J* = 8.3 Hz, *J* = 7.3 Hz, 1H, H_aromat._), 4.97 (d, *J* = 2.5 Hz, 1H, *H*C≡CCH_2_), 2.20 (t, *J* = 2.5 Hz, 2H, HC≡CC*H*_2_); ^13^C-NMR (151 MHz, DMSO-*d*_6_): δ = 162.45, 161.99, 146.30, 137.18, 134.69, 131.38, 130.52, 129.99, 129.76, 124.03, 123.66, 122.61, 79.37, 73.81, 29.90. Anal. Calcd. for C_15_H_8_N_2_O_4_: C, 64.29; H, 2.88; N, 10.00. Found: C, 64.33; H, 3.02; N, 9.95.

### 3.4. Synthesis of 5-Amino-2-(prop-2-yn-1-yl)-1H-benzo[de]isoquinoline-1,3(2H)-dione *(**12**)*

A suspension of compound **10** (1.00 mmol), propargyl amine (1.05 mmol) in ethanol (15 mL) was stirred at 78 °C for 3 h. The reaction mixture was cooled to room temperature and filtered to give **12** as orange needles which were pure to be used in the next step without further purification. Yield 92%; m.p. ˃250 °C; IR (KBr): ν = 3406, 3373, 3209, 2999, 1730, 1693, 1445, 782 cm^−1^; ^1^H-NMR (200 MHz, DMSO-*d*_6_): δ = 8.11 (d, *J* = 7.2 Hz, 1H, H_aromat._), 8.06 (d, *J* = 8.2 Hz, 1H, H_aromat._), 7.99 (d, *J* = 2.2 Hz, 1H, H_aromat._), 7.63 (dd, *J* = 8.2 Hz, *J* = 7.2 Hz, 1H, H_aromat._), 7.31 (d, *J* = 2.2 Hz, 1H, H_aromat._), 6.04 (s, 2H, NH_2_), 4.75 (d, *J* = 2.2 Hz, 1H, *H*C≡CCH_2_), 3.13 (t, *J* = 2.2 Hz, 2H, HC≡CC*H*_2_); ^13^C-NMR (151 MHz, DMSO-*d*_6_): δ = 163.48, 163.31, 148.42, 134.09, 132.41, 127.49, 126.20, 122.65, 122.38, 121.86, 120.97, 112.65, 80.00, 73.26, 29.47. Anal. Calcd. for C_15_H_10_N_2_O_2_: C, 71.99; H, 4.03; N, 11.19. Found: C, 71.74; H, 3.98; N, 10.96.

### 3.5. General Procedure for Copper(I)-Catalyzed Cycloaddition Reactions

To a solution of azidoalkylphosphonate **13a**–**i** (1.00 mmol) in EtOH (1.0 mL) and H_2_O (1.0 mL), CuSO_4_ × 5H_2_O (0.10 mmol), sodium ascorbate (0.20 mmol) and the respective *N*-propargyl naphthalimides **7/8**–**11/12** (1.00 mmol) were added. The reaction mixture was microwave irradiated at 35–40 °C for 15 min. After removal of solvents the residue was suspended in chloroform (5 mL), filtered through a layer of Celite and concentrated in vacuo. Crude products were purified on silica gel columns with chloroform–methanol mixtures (100:1 or 50:1, *v*/*v*) or crystallized from the appropriate solvents to give the 1,2,3-triazoles **14a**–**i**–**17a**–**i**.

*Diethyl {4-[1,3-Dioxo-1H-benzo[de]isoquinolin-2(3H)-yl)methyl]-1H-1,2,3-triazol-1-yl}methylphosphonate* (**14a**): Yield 81% (after crystallization from an ethyl acetate–hexane mixture). A white solid; m.p. 110–111 °C; IR (KBr): ν = 3446, 3245, 3234, 2983, 2931, 1700, 1659, 1236, 1023, 782, cm^−1^; ^1^H-NMR (200 MHz, CDCl_3_): δ = 8.54 (dd, *J* = 7.3 Hz, *J* = 1.2 Hz, 2H, H_aromat._), 8.16 (dd, *J* = 8.1 Hz, *J* = 1.2 Hz, 2H, H_aromat._), 7.86 (s, 1H, *H*C5′), 7.70 (dd, *J* = 8.1 Hz, *J* = 7.3 Hz, 2H, H_aromat._), 5.49 (s, 2H, CH_2_), 4.71 (d, *J* = 13.0 Hz, 2H, PCH_2_), 4.15–4.00 (m, 4H, 2 × POC*H*_2_CH_3_), 1.24 (t, *J* = 7.0 Hz, 6H, 2 × POCH_2_C*H*_3_); ^13^C-NMR (151 MHz, CDCl_3_): δ = 163.81, 144.18, 134.14, 131.63, 131.46, 128.24, 126.92, 124.27, 122.51, 63.46 (d, *J* = 6.6 Hz, POC), 45.83 (d, *J* = 155.3 Hz, PC), 35.18, 16.24 (d, *J* = 5.7 Hz, POC*C*); ^31^P-NMR (81 MHz, CDCl_3_): δ = 16.57 ppm. Anal. Calcd. for C_20_H_21_N_4_O_5_P: C, 56.08; H, 4.94; N, 13.08. Found: C, 56.21; H, 4.76; N, 12.84.

*Diethyl 2-{4-[(1,3-Dioxo-1H-benzo[de]isoquinolin-2(3H)-yl)methyl]-1H-1,2,3-triazol-1-yl}ethylphosphonate* (**14b**): Yield 79% (after crystallization from an ethyl acetate–hexane mixture). A white solid; m.p. 63–64 °C; IR (KBr): ν = 3422, 3245, 2981, 2927, 1701, 1659, 1588, 1237, 1026, 780 cm^−1^; ^1^H-NMR (200 MHz, CDCl_3_): δ = 8.62 (dd, *J* = 7.3 Hz, *J* = 1.2 Hz, 2H, H_aromat._), 8.22 (dd, *J* = 8.3 Hz, *J* = 1.2 Hz, 2H, H_aromat._), 7.75 (dd, *J* = 8.3 Hz, *J* = 7.3 Hz, 2H, H_aromat._), 7.69 (s, 1H, *H*C5′), 5.51 (s, 2H, CH_2_), 4.62–4.48 (m, 2H, PCH_2_C*H*_2_), 4.12–3.98 (m, 4H, 2 × POC*H*_2_CH_3_), 2.46–2.29 (m, 2H, PC*H*_2_), 1.26 (t, *J* = 7.0 Hz, 6H, 2 × POCH_2_C*H*_3_); ^13^C-NMR (151 MHz, CDCl_3_): δ = 163.85, 143.77, 134.78, 131.63, 131.49, 128.23, 126.95, 123.52, 122.51, 62.10 (d, *J* = 6.5 Hz, POC), 44.47 (PC*C*), 35.23, 27.29 (d, *J* = 143.0 Hz, PC), 16.32 (d, *J* = 5.9 Hz, POC*C*); ^31^P-NMR (81 MHz, CDCl_3_): δ = 26.30 ppm. Anal. Calcd. for C_21_H_23_N_4_O_5_P × 0.5H_2_O: C, 55.87; H, 5.36; N, 12.41. Found: C, 55.93; H, 5.15; N, 12.38.

*Diethyl 3-{4-[(1,3-Dioxo-1H-benzo[de]isoquinolin-2(3H)-yl)methyl]-1H-1,2,3-triazol-1-yl}propylphosphonate* (**14c**): Yield 78% (after crystallization from an ethyl acetate–hexane mixture). A white solid; m.p. 131–132 °C; IR (KBr): ν = 3440, 3143, 2984, 1703, 1662, 1590, 1236, 1050, 785, 755 cm^−1^; ^1^H-NMR (600 MHz, CDCl_3_): δ = 8.64 (d, *J* = 7.3 Hz, 2H, H_aromat._), 8.22 (d, *J* = 8.3 Hz, 2H, H_aromat._), 7.77 (dd, *J* = 8.3 Hz, *J* = 7.3 Hz, 2H, H_aromat._), 7.69 (s, 1H, *H*C5′), 5.53 (s, 2H, CH_2_), 4.41 (t, *J* = 6.9 Hz, 2H, PCH_2_CH_2_C*H*_2_), 4.12–4.03 (m, 4H, 2 × POC*H*_2_CH_3_), 2.20 (dqu, *J* = 21.7 Hz, *J* = 6.9 Hz, 2H, PCH_2_C*H*_2_), 1.72 (dt, *J* = 18.6 Hz, *J* = 8.0 Hz, 2H, PC*H*_2_), 1.30 (t, *J* = 7.1 Hz, 6H, 2 × POCH_2_C*H*_3_); ^13^C-NMR (151 MHz, CDCl_3_): δ = 163.84, 143.52, 134.12, 131.64, 131.48, 128.25, 126.92, 123.40, 122.55, 61.74 (d, *J* = 6.5 Hz, POC), 50.02 (d, *J* = 15.5 Hz, PCC*C*), 35.25, 23.65 (d, *J* = 4.5 Hz, PC*C*), 22.66 (d, *J* = 143.5 Hz, PC), 16.39 (d, *J* = 6.2 Hz, POC*C*); ^31^P-NMR (243 MHz, CDCl_3_): δ = 29.99 ppm. Anal. Calcd. for C_22_H_25_N_4_O_5_P: C, 57.89; H, 5.52; N, 12.27. Found: C, 57.73; H, 5.23; N, 12.26.

*Diethyl 4-{4-[(1,3-Dioxo-1H-benzo[de]isoquinolin-2(3H)-yl)methyl]-1H-1,2,3-triazol-1-yl}butylphosphonate* (**14d**): Yield 85% (after crystallization from an ethyl acetate–hexane mixture). A white solid; m.p. 131–132 °C; IR (KBr): ν = 3399, 3142, 3073, 2982, 2875, 1702, 1663, 1626, 1590, 1236, 1031, 786 cm^−1^; ^1^H-NMR (200 MHz, CDCl_3_): δ = 8.61 (dd, *J* = 7.3 Hz, *J* = 1.0 Hz, 2H, H_aromat._), 8.20 (d, *J* = 8.3 Hz, *J* = 1.0 Hz, 2H, H_aromat._), 7.74 (dd, *J* = 8.3 Hz, *J* = 7.3 Hz, 2H, H_aromat._), 7.63 (s, 1H, *H*C5′), 5.50 (s, 2H, CH_2_), 4.30 (t, *J* = 7.2 Hz, 2H, PCCCC*H*_2_), 4.13–3.95 (m, 4H, 2 × POC*H*_2_CH_3_), 2.00 (qu, *J* = 6.9 Hz, 2H, PCCC*H*_2_), 1.80–1.49 (m, 4H, PCC*H*_2_ and PC*H*_2_), 1.30 (t, *J* = 7.1 Hz, 6H, 2 × POCH_2_C*H*_3_); ^13^C-NMR (151 MHz, CDCl_3_): δ = 163.88, 143.73, 134.11, 131.63, 131.46, 128.25, 126.92, 123.13 122.56, 61.54 (d, *J* = 6.0 Hz, 2 × POC), 49.63, 35.27, 30.76 (d, *J* = 15.9 Hz, PCC*C*), 25.00 (d, *J* = 141.9 Hz, PC), 19.72 (d, *J* = 4.5 Hz, PC*C*), 16.41 (d, *J* = 6.0 Hz, POC*C*); ^31^P-NMR (81 MHz, CDCl_3_): δ = 31.78 ppm. Anal. Calcd. for C_23_H_27_N_4_O_5_P: C, 58.72; H, 5.78; N, 11.91. Found: C, 58.66; H, 5.64; N, 11.86.

*Diethyl 2-{4-[(1,3-Dioxo-1H-benzo[de]isoquinolin-2(3H)-yl)methyl]-1H-1,2,3-triazol-1-yl}-1-hydroxyethylphosphonate* (**14e**): Yield 80% (after crystallization from a methanol–diethyl ether mixture). A white solid; m.p. 186–188 °C; IR (KBr): ν = 3300, 3192, 2983, 1704, 1690, 1221, 1040, 1024, 787 cm^−1^; ^1^H-NMR (600 MHz, CDCl_3_): δ = 8.59 (dd, *J* = 7.3 Hz, *J* = 0.8 Hz, 2H, H_aromat._), 8.19 (dd, *J* = 8.2 Hz, *J* = 0.8 Hz, 2H, H_aromat._), 7.85 (s, 1H, *H*C5′), 7.75 (dd, *J* = 8.2 Hz, *J* = 7.3 Hz, 2H, H_aromat._), 5.51 (AB, *J* = 14.5 Hz, 1H, C*H*_a_H_b_), 5.49 (AB, *J* = 14.5 Hz, 1H, CH_a_*H*_b_), 4.78 (ddd, *J* = 14.3 Hz, *J* = 6.2 Hz, *J* = 2.5 Hz, 1H, PCC*H*_a_H_b_), 4.38 (ddd, *J* = 14.3 Hz, *J* = 9.6 Hz, *J* = 5.5 Hz, 1H, PCCH_a_*H*_b_), 4.35 (dt, *J* = 9.6 Hz, *J* = 2.5 Hz, 1H, PCH), 4.22–4.11 (m, 4H, 2 × POC*H*_2_CH_3_), 1.35 (t, *J* = 7.0 Hz, 3H, POCH_2_C*H*_3_), 1.32 (t, *J* = 7.0 Hz, 3H, POCH_2_C*H*_3_); ^13^C-NMR (151 MHz, CDCl_3_): δ = 163.74, 143.38, 134.04, 131.50, 131.35, 128.07, 126.87, 124.98, 122.2, 67.19 (d, *J* = 164.6 Hz, PC), 63.58 and 63.24 (2 × d, *J* = 7.6 Hz, 2 × POC), 51.57 (d, *J* = 9.1 Hz, PC*C*), 35.20, 16.42 and 16.38 (2 × d, *J* = 6.0 Hz, 2 × POC*C*); ^31^P-NMR (243 MHz, CDCl_3_): δ = 19.89 ppm. Anal. Calcd. for C_21_H_23_N_4_O_6_P: C, 55.02; H, 5.06; N, 12.22. Found: C, 55.10; H, 4.88; N, 12.22.

*Diethyl 3-{4-[(1,3-Dioxo-1H-benzo[de]isoquinolin-2(3H)-yl)methyl]-1H-1,2,3-triazol-1-yl}-2-hydroxypropylphosphonate* (**14f**): Yield 90% (after crystallization from a methanol–diethyl ether mixture). A white solid; m.p. >260 °C; IR (KBr): ν = 3430, 3148, 2986, 2930, 1776, 1740, 1237, 1029, 755 cm^−1^; ^1^H-NMR (200 MHz, CDCl_3_): δ = 8.60 (dd, *J* = 7.9 Hz, *J* = 0.9 Hz, 2H, H_aromat._), 8.20 (dd, *J* = 8.3 Hz, *J* = 0.9 Hz, 2H, H_aromat._), 7.82 (s, 1H, *H*C5′), 7.73 (dd, *J* = 7.9 Hz, *J* = 8.3 Hz, 2H, H_aromat._), 5.51 (s, 2H, CH_2_), 4.55–4.27 (m, 3H, PCCCH_2_, OH), 4.19–3.96 (m, 5H, PCCH, 2 × POC*H*_2_CH_3_), 2.06–1.67 (m, 2H, PCH_2_), 1.32 and 1.26 (t, *J* = 7.0 Hz, 3H, POCH_2_C*H*_3_); ^13^C-NMR (151 MHz, CDCl_3_): δ = 163.83, 143.63, 134.07, 131.59, 131.44, 128.21, 126.90, 124.85, 122.53, 65.62 (d, *J =* 3.0 Hz, PC*C*), 62.21 (d, *J* = 6.4 Hz, POC), 62.18 (d, *J* = 6.4 Hz, POC), 55.72 (d, *J* = 16.6 Hz, PCC*C*), 35.26, 30.59 (d, *J* = 139.8 Hz, PC), 16.30 (d, *J* = 6.0 Hz, 2 × POC*C*); ^31^P-NMR (81 MHz, CDCl_3_): δ = 29.28 ppm. Anal. Calcd. for C_22_H_25_N_4_O_6_P: C, 55.93; H, 5.33; N, 11.86. Found: C, 55.79; H, 5.14; N, 11.86.

*Diethyl 2-{4-[(1,3-Dioxo-1H-benzo[de]isoquinolin-2(3H)-yl)methyl]-1H-1,2,3-triazol-1-yl}ethoxy-methylphosphonate* (**14g**): Yield 82% (after crystallization from a methanol–diethyl ether mixture). A white solid; m.p. 154–155 °C; IR (KBr): ν = 3319, 3148, 3068, 3988, 2980, 2908, 1704, 1662, 1590, 1237, 1029, 784, 756 cm^−1^; ^1^H-NMR (200 MHz, CDCl_3_): δ = 8.60 (dd, *J* = 7.3 Hz, *J* = 1.0 Hz, 2H, H_aromat._), 8.19 (dd, *J* = 8.3 Hz, *J* = 1.0 Hz, 2H, H_aromat._), 7.77 (s, 1H, *H*C5′), 7.74 (dd, *J* = 8.3 Hz, *J* = 7.3 Hz, 2H, H_aromat._), 5.50 (s, 2H, CH_2_), 4.50 (t, *J* = 4.9 Hz, 2H, PCH_2_OCH_2_C*H*_2_), 4.15–4.00 (m, 4H, 2 × POC*H*_2_CH_3_) 3.95 (t, *J* = 4.9 Hz, 2H, PCOC*H*_2_CH_2_), 3.74 (d, *J* = 8.2 Hz, 2H, PCH_2_O), 1.28 (t, *J* = 7.1 Hz, 6H, 2 × POCH_2_C*H*_3_); ^13^C-NMR (151 MHz, CDCl_3_): δ = 163.82, 143.76, 134.09, 131.63, 131.43, 128.24, 126.92, 123.90, 122.57, 71.35 (d, *J* = 10.1 Hz, PCO*C*), 65.36 (d, *J* = 166.3 Hz, PC), 62.40 (d, *J* = 6.5 Hz, PO*C*), 50.02, 35.25, 16.42 (d, *J* = 6.0 Hz, 2 × POC*C*); ^31^P-NMR (81 MHz, CDCl_3_): δ = 21.16 ppm. Anal. Calcd. for C_22_H_25_N_4_O_6_P: C, 55.93; H, 5.33; N, 11.86. Found: C, 55.90; H, 5.28; N, 11.84.

*Diethyl 2-(2-{4-[(1,3-Dioxo-1H-benzo[de]isoquinolin-2(3H)-yl)methyl]-1H-1,2,3-triazol-1-yl}ethoxy)ethylphosphonate* (**14h**): Yield 79% (after crystallization from a methanol–diethyl ether mixture). A white solid; m.p. 156–158 °C; IR (KBr): ν = 3352, 3144, 2985, 2932, 2906, 1703, 1662, 1237, 1028, 785, 754 cm^−1^; ^1^H-NMR (200 MHz, CDCl_3_): δ = 8.60 (dd, *J* = 7.2 Hz, *J* = 0.9 Hz, 2H, H_aromat._), 8.22 (dd, *J* = 7.9 Hz, *J* = 0.9 Hz, 2H, H_aromat._), 7.78 (s, 1H, *H*C5′), 7.74 (dd, *J* = 7.9 Hz, *J* = 7.2 Hz, 1H, H_aromat._), 5.59 (s, 2H, CH_2_), 4.47 (t, *J* = 5.3 Hz, 2H, PCH_2_CH_2_OCH_2_C*H*_2_), 4.11–3.97 (m, 4H, 2 × POC*H*_2_CH_3_), 3.77 (t, *J* = 5.3 Hz, 2H, PCCOC*H*_2_CH_2_), 3.64 (dt, *J* = 11.8 Hz, *J* = 7.6 Hz, 2H, PCH_2_C*H*_2_O), 2.03 (dt, *J* = 18.7 Hz, *J* = 7.6 Hz, 2H, PC*H*_2_CH_2_O), 1.28 (t, *J* = 7.1 Hz, 6H, 2 × POCH_2_C*H*_3_); ^13^C-NMR (151 MHz, CDCl_3_): δ = 163.86, 143.56, 134.07, 131.63, 131.44, 128.25, 126.92, 124.28, 122.60, 68.90, 65.23 (PC*C*O), 61.65 (d, *J* = 6.0 Hz, PO*C*), 50.06, 35.27, 26.33 (d, *J* = 138.9 Hz, PC), 16.39 (d, *J* = 6.1 Hz, POC*C*); ^31^P-NMR (81 MHz, CDCl_3_): δ = 28.75 ppm. Anal. Calcd. for C_23_H_27_N_4_O_6_P: C, 56.79; H, 5.59; N, 11.52. Found: C, 56.72; H, 5.42; N, 11.70.

*Diethyl 2-{4-[(1,3-Dioxo-1H-benzo[de]isoquinolin-2(3H)-yl)methyl]-1H-1,2,3-triazol-1-yl}acetamido-methylphosphonate* (**14i**): Yield 91% (after column chromatography with chloroform–methanol mixtures (100:1 or 50:1, *v*/*v*)). A white powder; m.p. 170–171 °C; IR (KBr): ν = 3355, 2974, 2930, 1660, 1626, 1237, 1050, 782 cm^−1^; ^1^H-NMR (200 MHz, CDCl_3_): δ = 8.51 (dd, *J* = 7.3 Hz, *J* = 1.1 Hz, 2H, H_aromat._), 8.12 (dd, *J* = 8.2 Hz, *J* = 1.1 Hz, 2H, H_aromat._), 7.80 (s, 1H, *H*C5′), 7.66 (dd, *J* = 8.2 Hz, *J* = 7.3 Hz, 2H, H_aromat._), 7.49 (t, *J* = 5.9 Hz, 1H, NHCO), 5.44 (s, 2H, CH_2_), 5.01 (s, 2H), 4.06–3.92 (m, 4H, 2 × POC*H*_2_CH_3_), 3.62 (dd, *J* = 12.2 Hz, *J* = 5.9 Hz, 2H, PC*H*_2_NH), 1.17 (t, *J* = 7.1 Hz, 6H, 2 × POCH_2_C*H*_3_); ^13^C-NMR (151 MHz, CDCl_3_): δ = 165.24 (d, *J* = 5.5 Hz, C=O), 163.82, 144.09, 134.15, 131.60, 131.48, 128.20, 126.93, 124.93, 122.47, 62.84 (d, *J* = 6.5 Hz, PO*C*), 52.56, 35.17, 34.97 (d, *J* = 156.5 Hz, PC), 16.28 (d, *J* = 5.6 Hz, POC*C*); ^31^P-NMR (81 MHz, CDCl_3_): δ = 22.21 ppm. Anal. Calcd. for C_22_H_24_N_5_O_6_P: C, 54.43; H, 4.98; N, 14.43. Found: C, 54.38; H, 4.82; N, 14.35.

*Diethyl {4-[(6-Bromo-1,3-dioxo-1H-benzo[de]isoquinolin-2(3H)-yl)methyl]-1H-1,2,3-triazol-1-yl}methylphosphonate* (**15a**): Yield 82% (after column chromatography with chloroform–methanol mixtures (100:1 and 50:1, *v*/*v*)). A white powder; m.p. 158–159 °C; IR (KBr): ν = 3334, 3052, 3008, 2989, 2967, 1711, 1670, 1222, 1032, 757 cm^−1^; ^1^H-NMR (600 MHz, CDCl_3_): δ = 8.67 (d, *J* = 7.8 Hz, 1H, H_aromat._), 8.59 (d, *J* = 8.5 Hz, 1H, H_aromat._), 8.46 (d, *J* = 7.8 Hz, 1H, H_aromat._), 8.05 (d, *J* = 7.8 Hz, 1H, H_aromat._), 7.88 (s, 1H, *H*C5′), 7.86 (dd, *J* = 8.5 Hz, *J* = 7.8 Hz, 1H, H_aromat._), 5.53 (s, 2H, CH_2_), 4.74 (d, *J* = 13.0 Hz, 2H, PCH_2_), 4.15–4.00 (m, 4H, 2 × POC*H*_2_CH_3_), 1.29 (t, *J* = 7.0 Hz, 6H, 2 × POCH_2_C*H*_3_); ^13^C-NMR (151 MHz, CDCl_3_): δ = 163.24, 163.21, 143.87, 133.49, 132.28, 131.46, 131.13, 130.68, 130.52, 129.07, 128.08, 124.30, 122.94, 122.07, 63.45 (d, *J* = 6.4 Hz, POC), 45.63 (d, *J* = 156.4 Hz, PC), 35.26, 16.25 (d, *J* = 6.8 Hz, POC*C*); ^31^P-NMR (243 MHz, CDCl_3_): δ = 16.65 ppm. Anal. Calcd. for C_20_H_20_BrN_4_O_5_P: C, 47.35; H, 3.97; N, 11.04. Found: C, 47.31; H, 3.73; N, 10.99.

*Diethyl 2-{4-(6-Bromo-1,3-dioxo-1H-benzo[de]isoquinolin-2(3H)-yl)methyl)-1H-1,2,3-triazol-1-yl}ethylphosphonate* (**15b**): Yield 80% (after crystallization from ethyl acetate). A white solid; m.p. 132–134 °C; IR (KBr): ν = 3400, 3352, 3308, 2985, 2932, 1703, 1666, 1232, 1026, 779, 750 cm^−1^; ^1^H-NMR (200 MHz, CDCl_3_): δ = 8.61 (dd, *J* = 7.3 Hz, *J* = 1.2 Hz, 1H, H_aromat._), 8.50 (dd, *J* = 7.3 Hz, *J* = 1.2 Hz, 1H, H_aromat._), 8.37 (d, *J* = 8.3 Hz, 1H, H_aromat._), 7.98 (d, *J* = 7.9 Hz, 1H, H_aromat._), 7.78 (dd, *J* = 8.3 Hz, *J* = 7.3 Hz, 1H, H_aromat._), 7.64 (s, 1H, *H*C5′), 5.48 (s, 2H, CH_2_), 4.57–4.43 (m, 2H, PCH_2_C*H*_2_), 4.07–3.93 (m, 4H, 2 × POC*H*_2_CH_3_), 2.41–2.29 (m, 2H, PC*H*_2_), 1.21 (t, *J* = 7.1 Hz, 6H, 2 × POCH_2_C*H*_3_); ^13^C-NMR (151 MHz, CDCl_3_): δ = 163.15, 163.14, 143.43, 133.42, 132.22, 131.38, 131.09, 130.58, 130.48, 128.94, 128.06, 123.58, 122.85, 121.98, 62.09 (d, *J* = 6.5 Hz, POC), 44.48 (PC*C*), 35.28, 27.26 (d, *J* = 141.0 Hz, PC), 16.32 (d, *J* = 5.8 Hz, POC*C*); ^31^P-NMR (243 MHz, CDCl_3_): δ = 26.28 ppm. Anal. Calcd. for C_21_H_22_BrN_4_O_5_P: C, 48.38; H, 4.25; N, 10.75. Found: C, 48.07; H, 4.10; N, 10.62.

*Diethyl 3-{4-[(6-Bromo-1,3-dioxo-1H-benzo[de]isoquinolin-2(3H)-yl)methyl]-1H-1,2,3-triazol-1-yl}propylphosphonate* (**15c**): Yield 76% (after crystallization from an ethyl acetate–hexane mixture). A white solid; m.p. 118–120 °C; IR (KBr): ν = 3404, 2990, 2942, 2829, 1705, 1666, 1590, 1234, 1047, 1029, 752 cm^−1^; ^1^H-NMR (200 MHz, CDCl_3_): δ = 8.68 (dd, *J* = 7.3 Hz, *J* = 1.2 Hz, 1H, H_aromat._), 8.56 (dd, *J* = 8.3 Hz, *J* = 1.2 Hz, 1H, H_aromat._), 8.42 (d, *J* = 7.9 Hz, 1H, H_aromat._), 8.04 (d, *J* = 7.9 Hz, 1H, H_aromat._), 7.84 (dd, *J* = 8.3 Hz, *J* = 7.3 Hz, 1H, H_aromat._), 7.67 (s, 1H, *H*C5′), 5.49 (s, 2H, CH_2_), 4.40 (t, *J* = 7.0 Hz, 2H, PCH_2_CH_2_C*H*_2_), 4.15–3.96 (m, 4H, 2 × POC*H*_2_CH_3_), 2.18 (dqu, *J* = 21.0 Hz, *J* = 7.0 Hz, 2H, PCH_2_C*H*_2_), 1.72 (dt, *J* = 18.9 Hz, *J* = 8.0 Hz, 2H, PC*H*_2_), 1.28 (t, *J* = 7.1 Hz, 6H, 2 × POCH_2_C*H*_3_); ^13^C-NMR (151 MHz, CDCl_3_): δ = 163.29, 163.26, 143.50, 133.49, 132.31, 131.47, 131.12, 130.68, 130.51, 129.09, 128.08, 123.48, 122.97, 122.10, 61.76 (d, *J* = 6.5 Hz, POC), 50.02 (d, *J* = 15.1 Hz, PCC*C*), 35.32, 23.64 (d, *J* = 4.5 Hz, PC*C*), 22.66 (d, *J* = 143.5 Hz, PC), 16.40 (d, *J* = 5.7 Hz, POC*C*); ^31^P-NMR (243 MHz, CDCl_3_): δ = 30.85 ppm. Anal. Calcd. for C_22_H_24_BrN_4_O_5_P: C, 49.36; H, 4.52; N, 10.47. Found: C, 49.25; H, 4.44; N, 10.45.

*Diethyl 4-{4-[(6-Bromo-1,3-dioxo-1H-benzo[de]isoquinolin-2(3H)-yl)methyl]-1H-1,2,3-triazol-1-yl}butylphosphonate* (**15d**): Yield 88% (after crystallization from an ethyl acetate–hexane mixture). A white solid; m.p. 110–112 °C; IR (KBr): ν = 3357, 2982, 2938, 2909, 2875, 1794, 1703, 1024, 962 cm^−1^; ^1^H-NMR (200 MHz, CDCl_3_): δ = 8.67 (dd, *J* = 7.3 Hz, *J* = 1.2 Hz, 1H, H_aromat._), 8.57 (d, *J* = 8.3 Hz, *J* = 1.2 Hz, 1H, H_aromat._), 8.42 (d, *J* = 7.9 Hz, 1H, H_aromat._), 8.04 (d, *J* = 7.9 Hz, 1H, H_aromat._), 7.84 (dd, *J* = 8.3 Hz, *J* = 7.3 Hz, 1H, H_aromat._), 7.63 (s, 1H, *H*C5′), 5.48 (s, 2H, CH_2_), 4.31 (t, *J* = 7.2 Hz, 2H, PCCCC*H*_2_), 4.12–3.97 (m, 4H, 2 × POC*H*_2_CH_3_), 2.12–1.90 (m, 2H, PCCC*H*_2_), 1.84–1.49 (m, 4H, PCC*H*_2_ and PC*H*_2_), 1.30 (t, *J* = 7.2 Hz, 6H, 2 × POCH_2_C*H*_3_); ^13^C-NMR (151 MHz, CDCl_3_): δ = 163.25, 163.22, 143.41, 133.43, 132.26, 131.43, 131.09, 130.65, 130.50, 129.05, 128.06, 123.17, 122.96, 122.08, 61.54 (d, *J* = 7.6 Hz, 2 × POC), 49.64, 35.33, 30.74 (d, *J* = 13.6 Hz, PCC*C*), 24.95 (d, *J* = 143.5 Hz, PC), 19.71 (d, *J* = 4.5 Hz, PC*C*), 16.40 (d, *J* = 6.0 Hz, POC*C*); ^31^P-NMR (81 MHz, CDCl_3_): δ = 31.77 ppm. Anal. Calcd. for C_23_H_26_BrN_4_O_5_P: C, 50.29; H, 4.77; N, 10.20. Found: C, 50.11; H, 4.62; N, 10.00.

*Diethyl 2-{4-[(6-Bromo-1,3-dioxo-1H-benzo[de]isoquinolin-2(3H)-yl)methyl]-1H-1,2,3-triazol-1-yl}-1-hydroxyethylphosphonate* (**15e**): Yield 80% (after crystallization from an ethyl acetate–hexane mixture). A white solid; m.p. 198–200 °C; IR (KBr): ν = 3261, 2987, 2933, 2909, 1704, 1665, 1234, 1046, 1023, 753 cm^−1^; ^1^H-NMR (200 MHz, CDCl_3_): δ = 8.50 (dd, *J* = 7.3 Hz, *J* = 1.1 Hz, 1H, H_aromat._), 8.44 (d, *J* = 8.3 Hz, *J* = 1.1 Hz, 1H, H_aromat._), 8.26 (d, *J* = 7.9 Hz, 1H, H_aromat._), 7.94 (d, *J* = 7.9 Hz, 1H, H_aromat._), 7.88 (s, 1H, *H*C5′), 7.75 (dd, *J* = 8.3 Hz, *J* = 7.3 Hz, 1H, H_aromat._), 5.41 (s, 2H, CH_2_), 4.79 (ddd, *J* = 11.3 Hz, *J* = 6.3 Hz, *J* = 2.3 Hz, 1H, PCC*H*_a_H_b_), 4.46 (dt, *J* = 9.9 Hz, *J* = 2.3 Hz, 1H, PCH), 4.38 (ddd, *J* = 11.3 Hz, *J* = 9.9 Hz, *J* = 5.0 Hz, 1H, PCCH_a_*H*_b_), 4.21–4.06 (m, 4H, 2 × POC*H*_2_CH_3_), 1.31 (t, *J* = 6.9 Hz, 3H, POCH_2_C*H*_3_), 1.29 (t, *J* = 6.9 Hz, 3H, POCH_2_C*H*_3_); ^13^C-NMR (151 MHz, CDCl_3_): δ = 163.09, 163.00, 143.05, 133.36, 132.15, 131.31, 131.07, 130.45, 128.78, 128.02, 126.87, 125.10, 122.73, 121.87, 67.20 (d, *J* = 164.6 Hz, PC), 63.40 (d, *J* = 7.6 Hz, POC), 63.25 (d, *J* = 7.6 Hz, POC), 51.64 (d, *J* = 9.1 Hz, PC*C*), 35.27, 16.44 (d, *J* = 6.0 Hz, POC*C*), 16.40 (d, *J* = 6.0 Hz, POC*C*); ^31^P-NMR (81 MHz, CDCl_3_): δ = 20.91 ppm. Anal. Calcd. for C_21_H_22_BrN_4_O_6_P: C, 46.94; H, 4.13; N, 10.43. Found: C, 47.07; H, 3.91; N, 10.48.

*Diethyl 3-{4-[(6-Bromo-1,3-dioxo-1H-benzo[de]isoquinolin-2(3H)-yl)methyl]-1H-1,2,3-triazol-1-yl}-2-hydroxypropylphosphonate* (**15f**): Yield 85% (after crystallization from a methanol–diethyl ether mixture). A white solid; m.p. 152–153 °C; IR (KBr): ν = 3330, 3155, 2986, 2909, 1704, 1664, 1234, 1027, 752 cm^−1^; ^1^H-NMR (200 MHz, CDCl_3_): δ = 8.54 (dd, *J* = 7.5 Hz, *J* = 1.0 Hz, 1H, H_aromat._), 8.48 (d, *J* = 8.5 Hz, *J* = 1.0 Hz, 1H, H_aromat._), 8.29 (d, *J* = 7.9 Hz, 1H, H_aromat._), 7.96 (d, *J* = 7.9 Hz, 1H, H_aromat._), 7.90 (s, 1H, *H*C5′), 7.65 (dd, *J* = 8.5 Hz, *J* = 7.5 Hz, 1H, H_aromat._), 5.49 (s, 2H, CH_2_), 4.52–4.17 (m, 3H, PCCCH_2_, OH), 4.16–3.95 (m, 5H, PCCH, 2 × POC*H*_2_CH_3_), 2.10–1.60 (m, 2H, PCH_2_), 1.33 and 1.29 (t, *J* = 7.0 Hz, 3H, POCH_2_C*H*_3_); ^13^C-NMR (151 MHz, CDCl_3_): δ = 163.30, 163.28, 143.33, 133.47, 132.31, 131.47, 131.12, 130.64, 130.50, 129.05, 128.07, 124.95, 122.94, 122.07, 65.61 (d, *J =* 4.4 Hz, PC*C*), 62.30 (d, *J* = 6.0 Hz, POC), 62.20 (d, *J* = 6.0 Hz, POC), 55.75 (d, *J* = 17.6 Hz, PCC*C*), 35.34, 30.59 (d, *J* = 141.8 Hz, PC), 16.30 (d, *J* = 6.0 Hz, 2 × POC*C*); ^31^P-NMR (81 MHz, CDCl_3_): δ = 29.21 ppm. Anal. Calcd. for C_22_H_24_BrN_4_O_6_P: C, 47.93; H, 4.39; N, 10.16. Found: C, 47.94; H, 4.50; N, 10.16.

*Diethyl 2-{4-[(6-Bromo-1,3-dioxo-1H-benzo[de]isoquinolin-2(3H)-yl)methyl]-1H-1,2,3-triazol-1-yl}ethoxy-methylphosphonate* (**15g**): Yield 90% (after crystallization from an ethyl acetate–hexane mixture). A white solid; m.p. 127–128 °C; IR (KBr): ν = 3441, 3148, 3087, 2985, 2935, 2908, 1704, 1665, 1234, 1027, 751 cm^−1^; ^1^H-NMR (200 MHz, CDCl_3_): δ = 8.51 (dd, *J* = 7.3 Hz, *J* = 1.2 Hz, 1H, H_aromat._), 8.47 (d, *J* = 8.3 Hz, *J* = 1.2 Hz, 1H, H_aromat._), 8.45 (d, *J* = 7.9 Hz, 1H, H_aromat._), 7.99 (d, *J* = 7.9 Hz, 1H, H_aromat._), 7.74 (dd, *J* = 8.3 Hz, *J* = 7.3 Hz, 1H, H_aromat._), 7.70 (s, 1H, *H*C5′), 5.49 (s, 2H, CH_2_), 4.49 (t, *J* = 5.1 Hz, 2H, PCH_2_OCH_2_C*H*_2_), 4.18–3.98 (m, 4H, 2 × POC*H*_2_CH_3_), 3.94 (t, *J* = 5.1 Hz, 2H, PCOC*H*_2_CH_2_), 3.70 (d, *J* = 8.1 Hz, 2H, PCH_2_O), 1.30 (t, *J* = 7.2 Hz, 6H, 2 × POCH_2_C*H*_3_); ^13^C-NMR (151 MHz, CDCl_3_): δ = 163.25, 163.23, 143.32, 133.44, 132.26, 131.43, 131.19, 130.67, 130.45, 129.08, 128.08, 124.21, 123.00, 122.13, 71.32 (d, *J* = 9.1 Hz, PCO*C*), 65.16 (d, *J* = 166.1 Hz, PC), 62.47 (d, *J* = 6.3 Hz, PO*C*), 50.03, 35.32, 16.44 (d, *J* = 5.4 Hz, 2 × POC*C*); ^31^P-NMR (81 MHz, CDCl_3_): δ = 21.17 ppm. Anal. Calcd. for C_22_H_24_BrN_4_O_6_P: C, 47.93; H, 4.39; N, 10.16. Found: C, 48.07; H, 4.10; N, 9.97.

*Diethyl 2-(2-{4-[(6-Bromo-1,3-dioxo-1H-benzo[de]isoquinolin-2(3H)-yl)methyl]-1H-1,2,3-triazol-1-yl}ethoxy)ethylphosphonate* (**15h**): Yield 86% (after crystallization from an ethyl acetate–hexane mixture). A white solid; m.p. 92–93 °C; IR (KBr): ν = 3145, 3086, 2984, 2930, 2907, 2876, 1704, 1666, 1234, 1047, 751 cm^−1^; ^1^H-NMR (200 MHz, CDCl_3_): δ = 8.72 (dd, *J* = 7.3 Hz, *J* = 1.0 Hz, 1H, H_aromat._), 8.55 (d, *J* = 8.3 Hz, *J* = 1.0 Hz, 1H, H_aromat._), 8.49 (d, *J* = 7.9 Hz, 1H, H_aromat._), 8.10 (d, *J* = 7.9 Hz, 1H, H_aromat._), 7.84 (dd, *J* = 8.3 Hz, *J* = 7.3 Hz, 1H, H_aromat._), 7.76 (s, 1H, *H*C5′), 5.52 (s, 2H, CH_2_), 4.49 (t, *J* = 5.3 Hz, 2H, PCH_2_CH_2_OCH_2_C*H*_2_), 4.18–3.95 (m, 4H, 2 × POC*H*_2_CH_3_), 3.79 (t, *J* = 5.3 Hz, 2H, PCCOC*H*_2_CH_2_), 3.56 (dt, *J* = 12.0 Hz, *J* = 7.5 Hz, 2H, PCH_2_C*H*_2_O), 2.03 (dt, *J* = 18.4 Hz, *J* = 7.5 Hz, 2H, PC*H*_2_CH_2_O), 1.25 (t, *J* = 6.9 Hz, 6H, 2 × POCH_2_C*H*_3_); ^13^C-NMR (151 MHz, CDCl_3_): δ = 163.27, 163.25, 143.28, 133.40, 132.26, 131.43, 131.11, 130.66, 130.42, 129.09, 128.07, 124.31, 123.03, 122.16, 68.97, 65.24 (PC*C*O), 61.63 (d, *J* = 5.7 Hz, PO*C*), 50.08, 35.36, 26.10 (d, *J* = 140.1 Hz, PC), 16.40 (d, *J* = 6.0 Hz, POC*C*); ^31^P-NMR (81 MHz, CDCl_3_): δ = 28.76 ppm. Anal. Calcd. for C_23_H_26_BrN_4_O_6_P: C, 48.86; H, 4.64; N, 9.91. Found: C, 48.83; H, 4.56; N, 10.10.

*Diethyl 2-{4-[(6-Bromo-1,3-dioxo-1H-benzo[de]isoquinolin-2(3H)-yl)methyl]-1H-1,2,3-triazol-1-yl}acetamido-methylphosphonate* (**15i**): Yield 88% (after column chromatography with chloroform–methanol mixtures (100:1 or 50:1, *v*/*v*)). A white solid; m.p. 173–174 °C; IR (KBr): ν = 3240, 3148, 3071, 2987, 2932, 1703, 1665, 1234, 1025, 752 cm^−1^; ^1^H-NMR (200 MHz, CDCl_3_): δ = 8.52 (dd, *J* = 7.3 Hz, *J* = 0.8 Hz, 1H, H_aromat._), 8.42 (d, *J* = 8.3 Hz, *J* = 0.8 Hz, 1H, H_aromat._), 8.39 (d, *J* = 7.9 Hz, 1H, H_aromat._), 8.00 (d, *J* = 7.9 Hz, 1H, H_aromat._), 7.84 (s, 1H, *H*C5′), 7.80 (dd, *J* = 8.3 Hz, *J* = 7.3 Hz, 1H, H_aromat._), 7.40 (brt, *J* = 5.9 Hz, 1H, NHCO), 5.56 (s, 2H, CH_2_), 5.01 (s, 2H), 4.16–3.98 (m, 4H, 2 × POC*H*_2_CH_3_), 3.62 (dd, *J* = 12.4 Hz, *J* = 6.0 Hz, 2H, PC*H*_2_NH), 1.12 (t, *J* = 7.2 Hz, 6H, 2 × POCH_2_C*H*_3_); ^13^C-NMR (151 MHz, CDCl_3_): δ = 165.26 (d, *J* = 5.5 Hz, C=O), 163.25, 163.24, 143.76, 133.49, 132.29, 131.45, 131.12, 130.62, 130.54, 128.99, 128.07, 125.02, 122.85, 121.98, 62.87 (d, *J* = 6.6 Hz, PO*C*), 52.57, 35.25, 35.00 (d, *J* = 155.4 Hz, PC), 16.30 (d, *J* = 5.4 Hz, POC*C*); ^31^P-NMR (81 MHz, CDCl_3_): δ = 22.29 ppm. Anal. Calcd. for C_22_H_23_BrN_5_O_6_P: C, 46.82; H, 4.11; N, 12.41. Found: C, 46.62; H, 3.90; N, 12.11.

*Diethyl {4-[(5-Nitro-1,3-dioxo-1H-benzo[de]isoquinolin-2(3H)-yl)methyl]-1H-1,2,3-triazol-1-yl}methylphosphonate* (**16a**): Yield 71% (after crystallization from an ethyl acetate–hexane mixture). A white solid; m.p. 147–148 °C; IR (KBr): ν = 3335, 2988, 2939, 1698, 1711, 1670, 1244, 1110, 790, 757 cm^−1^; ^1^H-NMR (200 MHz, CDCl_3_): δ = 9.24 (d, *J* = 2.2 Hz, 1H, H_aromat._), 9.07 (d, *J* = 2.2 Hz, 1H, H_aromat._), 8.73 (dd, *J* = 7.4 Hz, *J* = 1.2 Hz, 1H, H_aromat._), 8.36 (dd, *J* = 8.4 Hz, *J* = 1.2 Hz, 1H, H_aromat._), 7.88 (dd, *J* = 8.4 Hz, *J* = 7.4 Hz, 1H, H_aromat._), 7.84 ( s, 1H, *H*C5′), 5.47 (s, 2H, CH_2_), 4.66 (d, *J* = 13.1 Hz, 2H, PCH_2_), 4.13–3.98 (m, 4H, 2 × POC*H*_2_CH_3_), 1.22 (2 × t, *J* = 7.0 Hz, 6H, 2 × POCH_2_C*H*_3_); ^13^C-NMR (151 MHz, CDCl_3_): δ = 162.70, 162.17, 146.36, 143.43, 135.78, 134.68, 131.05, 130.23, 129.12, 129.11, 124.52, 124.39, 124.35, 123.04, 63.50 (d, *J* = 6.6 Hz, POC), 45.36 (d, *J* = 155.6 Hz, PC), 35.47, 16.27 (d, *J* = 5.6 Hz, POC*C*); ^31^P-NMR (81 MHz, CDCl_3_): δ = 16.50 ppm. Anal. Calcd. for C_20_H_20_N_5_O_7_P: C, 50.74; H, 4.26; N, 14.79. Found: C, 50.73; H, 3.99; N, 14.93.

*Diethyl 2-{4-[(5-Nitro-1,3-dioxo-1H-benzo[de]isoquinolin-2(3H)-yl)methyl]-1H-1,2,3-triazol-1-yl}ethylphosphonate* (**16b**): Yield 88% (after crystallization from an ethyl acetate–hexane mixture). White needles; m.p. 170–171 °C; IR (KBr): ν = 3284, 3142, 3079, 2934, 2910, 1713, 1667, 1244, 790, 703 cm^−1^; ^1^H-NMR (200 MHz, CDCl_3_): δ = 9.26 (d, *J* = 2.2 Hz, 1H, H_aromat._), 9.08 (d, *J* = 2.2 Hz, 1H, H_aromat._), 8.74 (dd, *J* = 7.3 Hz, *J* = 1.1 Hz, 1H, H_aromat._), 8.38 (dd, *J* = 8.3 Hz, *J* = 1.1 Hz, 1H, H_aromat._), 7.89 (dd, *J* = 8.3 Hz, *J* = 7.3 Hz, 1H, H_aromat._), 7.68 (s, 1H, *H*C5′), 5.46 (s, 2H, CH_2_), 4.58–4.45 (m, 2H, PCH_2_C*H*_2_), 4.09–3.94 (m, 4H, 2 × POC*H*_2_CH_3_), 2.42‒2.24 (m, 2H, PC*H*_2_), 1.23 (t, *J* = 7.1 Hz, 6H, 2 × POCH_2_C*H*_3_); ^13^C-NMR (151 MHz, CDCl_3_): δ = 162.74, 162.23, 146.40, 143.04, 135.77, 134.72, 131.06, 130.25, 129.13, 129.13, 124.54, 124.46, 123.61, 123.07, 62.14 (d, *J* = 6.0 Hz, POC), 44.55 (PC*C*), 35.50, 27.30 (d, *J* = 140.4 Hz, PC), 16.34 (d, *J* = 5.7 Hz, POC*C*); ^31^P-NMR (81 MHz, CDCl_3_): δ = 26.23 ppm. Anal. Calcd. for C_21_H_22_N_4_O_7_P: C, 51.75; H, 4.55; N, 14.37. Found: C, 51.54; H, 4.43; N, 14.17.

*Diethyl 3-{4-[(5-Nitro-1,3-dioxo-1H-benzo[de]isoquinolin-2(3H)-yl)methyl]-1H-1,2,3-triazol-1-yl}propylphosphonate* (**16c**): Yield 80% (after crystallization from ethyl acetate). A white solid; m.p. 113–114 °C; IR (KBr): ν = 3404, 3084, 2982, 2943, 1712, 1671, 1597, 1244, 1112, 1029, 791, 758 cm^−1^; ^1^H-NMR (200 MHz, CDCl_3_): δ = 9.32 (d, *J* = 2.1 Hz, 1H, H_aromat._), 9.13 (d, *J* = 2.1 Hz, 1H, H_aromat._), 8.80 (dd, *J* = 7.3 Hz, *J* = 1.1 Hz, 1H, H_aromat._), 8.43 (dd, *J* = 8.3 Hz, *J* = 1.1 Hz, 1H, H_aromat._), 7.94 (dd, *J* = 8.3 Hz, *J* = 7.3 Hz, 1H, H_aromat._), 7.71 (s, 1H, *H*C5′), 5.52 (s, 2H, CH_2_), 4.41 (t, *J* = 7.0 Hz, 2H, PCH_2_CH_2_C*H*_2_), 4.15–3.97 (m, 4H, 2 × POC*H*_2_CH_3_), 2.30–2.08 (m, 2H, PCH_2_C*H*_2_), ), 1.80–1.60 (m, 2H, PC*H*_2_), 1.29 (t, *J* = 7.0 Hz, 6H, 2 × POCH_2_C*H*_3_); ^13^C-NMR (151 MHz, CDCl_3_): δ = 162.76, 162.25, 146.37, 143.06, 135.77, 134.74, 131.07, 130.26, 129.11, 129.11, 124.56, 124.43, 123.39, 123.08, 61.79 (d, *J* = 6.0 Hz, POC), 50.00 (d, *J* = 15.1 Hz, PCC*C*), 35.50, 23.64 (d, *J* = 4.4 Hz, PC*C*), 22.46 (d, *J* = 143.5 Hz, PC), 16.40 (d, *J* = 5.4 Hz, POC*C*); ^31^P-NMR (81 MHz, CDCl_3_): δ = 30.84 ppm. Anal. Calcd. for C_22_H_24_N_5_O_7_P: C, 52.70; H, 4.82; N, 13.97. Found: C, 52.75; H, 4.93; N, 14.01.

*Diethyl 4-{4-[(5-Nitro-1,3-dioxo-1H-benzo[de]isoquinolin-2(3H)-yl)methyl]-1H-1,2,3-triazol-1-yl}butylphosphonate* (**16d**): Yield 82% (after crystallization from ethyl an acetate–hexane mixture). White needles; m.p. 150–151 °C; IR (KBr): ν = 3369, 3145, 3082, 2987, 1711, 1670, 1243, 1027, 791, 754 cm^−1^; ^1^H-NMR (200 MHz, CDCl_3_): δ = 9.31 (d, *J* = 2.1 Hz, 1H, H_aromat._), 9.13 (d, *J* = 2.1 Hz, 1H, H_aromat._), 8.79 (dd, *J* = 7.4 Hz, *J* = 1.1 Hz, 1H, H_aromat._), 8.42 (dd, *J* = 8.3 Hz, *J* = 1.1 Hz, 1H, H_aromat._), 7.94 (dd, *J* = 8.3 Hz, *J* = 7.4 Hz, 1H, H_aromat._), 7.67 (s, 1H, *H*C5′), 5.52 (s, 2H, CH_2_), 4.30 (t, *J* = 7.1 Hz, 2H, PCCCC*H*_2_), 4.13–3.95 (m, 4H, 2 × POC*H*_2_CH_3_), 2.07–1.92 (m, 2H, PCCC*H*_2_), 1.82–1.53 (m, 4H, PCC*H*_2_ and PC*H*_2_), 1.28 (t, *J* = 7.1 Hz, 6H, 2 × POCH_2_C*H*_3_); ^13^C-NMR (151 MHz, CDCl_3_): δ = 162.75, 162.25, 146.38, 142.99, 135.73, 134.72, 131.06, 130.27, 129.11, 129.09, 124.59, 124.44, 123.28, 123.11, 61.59 (d, *J* = 6.6 Hz, 2 × POC), 49.71, 35.52, 30.76 (d, *J* = 15.8 Hz, PCC*C*), 25.00 (d, *J* = 141.9 Hz, PC), 19.72 (d, *J* = 4.9 Hz, PC*C*), 16.41 (d, *J* = 6.0 Hz, POC*C*); ^31^P-NMR (81 MHz, CDCl_3_): δ = 31.73 ppm. Anal. Calcd. for C_23_H_26_N_5_O_7_P: C, 53.59; H, 5.08; N, 13.59. Found: C, 53.56; H, 4.92; N, 13.55.

*Diethyl 2-{4-[(5-Nitro-1,3-dioxo-1H-benzo[de]isoquinolin-2(3H)-yl)methyl]-1H-1,2,3-triazol-1-yl}-1-hydroxyethylphosphonate* (**16e**): Yield 83% (after crystallization from an ethyl acetate–hexane mixture). A white solid; m.p. 212–213 °C; IR (KBr): ν = 3302, 3130, 2973, 1710, 1694, 1228, 1027, 789 cm^−1^; ^1^H-NMR (200 MHz, CDCl_3_): δ = 9.30 (d, *J* = 2.2 Hz, 1H, H_aromat._), 9.13 (d, *J* = 2.2 Hz, 1H, H_aromat._), 8.79 (dd, *J* = 7.3 Hz, *J* = 1.2 Hz, 1H, H_aromat._), 8.42 (dd, *J* = 8.3 Hz, *J* = 1.2 Hz, 1H, H_aromat._), 7.94 (dd, *J* = 8.3 Hz, *J* = 7.3 Hz, 1H, H_aromat._), 7.85 (s, 1H, *H*C5′), 5.52 (AB, *J* = 14.2 Hz, 1H, C*H_a_*H_b_), 5.49 (AB, *J* = 14.2 Hz, 1H, CH_a_*H_b_*), 4.76 (ddd, *J* = 13.8 Hz, *J* = 6.8 Hz, *J* = 2.6 Hz, 1H, PCC*H*_a_H_b_), 4.52–4.27 (m, 2H, PCCH_a_*H*_b_, PCH), 4.26–4.08 (m, 4H, 2 × POC*H*_2_CH_3_), 1.34 (t, *J* = 7.0 Hz, 3H, POCH_2_C*H*_3_), 1.32 (t, *J* = 7.0 Hz, 3H, POCH_2_C*H*_3_); ^13^C-NMR (151 MHz, CDCl_3_): δ = 162.74, 162.21, 146.38, 142.78, 135.73, 134.70, 131.04, 130.23, 129.12, 129.08, 124.98, 124.55, 124.41, 123.08, 67.15 (d, *J* = 164.6 Hz, PC), 63.46 (d, *J* = 6.6 Hz, POC), 63.24 (d, *J* = 6.6 Hz, POC), 51.55 (d, *J* = 9.1 Hz, PC*C*), 35.51, 16.44 (d, *J* = 6.0 Hz, POC*C*), 16.40 (d, *J* = 6.0 Hz, POC*C*); ^31^P-NMR (81 MHz, CDCl_3_): δ = 20.63 ppm. Anal. Calcd. for C_21_H_22_N_5_O_8_P: C, 50.10; H, 4.40; N, 13.91. Found: C, 55.02; H, 4.14; N, 13.86.

*Diethyl 3-{4-[(5-Nitro-1,3-dioxo-1H-benzo[de]isoquinolin-2(3H)-yl)methyl]-1H-1,2,3-triazol-1-yl}-2-hydroxypropylphosphonate* (**16f**): Yield 86% (after crystallization from an ethyl acetate–hexane mixture). A white solid; m.p. 176–177 °C; IR (KBr): ν = 3279, 3135, 3080, 2986, 2931, 2830, 1709, 1667, 1232, 1033, 799, 755 cm^−1^; ^1^H-NMR (200 MHz, CDCl_3_): δ = 9.31 (d, *J* = 2.2 Hz, 1H, H_aromat._), 9.13 (d, *J* = 2.2 Hz, 1H, H_aromat._), 8.79 (dd, *J* = 7.4 Hz, *J* = 1.2 Hz, 1H, H_aromat._), 8.43 (dd, *J* = 8.2 Hz, *J* = 1.2 Hz, 1H, H_aromat._), 7.94 (dd, *J* = 8.2 Hz, *J* = 7.4 Hz, 1H, H_aromat._), 7.86 (s, 1H, *H*C5′), 5.53 (s, 2H, CH_2_), 4.56–4.31 (m, 3H, PCCCH_2_, OH), 4.18–4.00 (m, 5H, PCCH, 2 × POC*H*_2_CH_3_), 2.06–1.74 (m, 2H, PCH_2_), 1.31 and 1.30 (t, *J* = 7.0 Hz, 3H, POCH_2_C*H*_3_); ^13^C-NMR (151 MHz, CDCl_3_): δ = 162.75, 162.22, 146.39, 142.92, 135.70, 134.70, 131.06, 130.27, 129.09, 129.06, 124.96, 124.60, 124.44, 123.12, 65.59 (d, *J =* 3.0 Hz, PC*C*), 62.32 (d, *J* = 6.4 Hz, POC), 62.26 (d, *J* = 6.4 Hz, POC), 55.75 (d, *J* = 16.6 Hz, PCC*C*), 35.55, 30.56 (d, *J* = 140.4 Hz, PC), 16.36 (d, *J* = 6.0 Hz, POC*C*), 16.32 (d, *J* = 6.0 Hz, POC*C*); ^31^P-NMR (81 MHz, CDCl_3_): δ = 29.28 ppm. Anal. Calcd. for C_22_H_24_N_5_O_8_P: C, 51.07; H, 4.68; N, 13.53. Found: C, 51.18; H, 4.43; N, 13.77.

*Diethyl 2-{4-[(5-Nitro-1,3-dioxo-1H-benzo[de]isoquinolin-2(3H)-yl)methyl]-1H-1,2,3-triazol-1-yl}ethoxy-methylphosphonate* (**16g**): Yield 82% (after crystallization from an ethyl acetate–hexane mixture). A yellow powder; m.p. 89–90 °C; IR (KBr): ν = 3397, 3019, 1712, 1672, 1215, 1048, 757 cm^−1^; ^1^H-NMR (200 MHz, CDCl_3_): δ = 9.30 (d, *J* = 2.2 Hz, 1H, H_aromat._), 9.12 (d, *J* = 2.2 Hz, 1H, H_aromat._), 8.78 (dd, *J* = 7.3 Hz, *J* = 1.2 Hz, 1H, H_aromat._), 8.44 (dd, *J* = 8.3 Hz, *J* = 1.2 Hz, 1H, H_aromat._), 7.94 (dd, *J* = 8.3 Hz, *J* = 7.3 Hz, 1H, H_aromat._), 7.82 (s, 1H, *H*C5′), 5.51 (s, 2H, CH_2_), 4.52 (t, *J* = 4.8 Hz, 2H, PCH_2_OCH_2_C*H*_2_), 4.18–4.04 (m, 4H, 2 × POC*H*_2_CH_3_) 3.95 (t, *J* = 4.8 Hz, 2H, PCOC*H*_2_CH_2_), 3.75 (d, *J* = 8.2 Hz, 2H, PCH_2_O), 1.31 (t, *J* = 7.1 Hz, 6H, 2 × POCH_2_C*H*_3_); ^13^C-NMR (151 MHz, CDCl_3_): δ = 162.72, 162.21, 146.36, 142.92, 135.72, 134.67, 131.04, 130.26, 129.10, 129.07, 124.60, 124.37, 124.31, 123.11, 71.32 (d, *J* = 10.6 Hz, PCO*C*), 65.34 (d, *J* = 166.1 Hz, PC), 62.50 (d, *J* = 6.5 Hz, PO*C*), 50.04, 35.52, 16.46 (d, *J* = 6.0 Hz, 2 × POC*C*); ^31^P-NMR (81 MHz, CDCl_3_): δ = 21.15 ppm. Anal. Calcd. for C_22_H_24_N_5_O_8_P: C, 51.07; H, 4.68; N, 13.53. Found: C, 51.10; H, 4.39; N, 13.62.

*Diethyl 2-(2-{4-[(5-Nitro-1,3-dioxo-1H-benzo[de]isoquinolin-2(3H)-yl)methyl]-1H-1,2,3-triazol-1-yl}ethoxy)ethylphosphonate* (**16h**): Yield 89%; (after column chromatography with chloroform–methanol mixtures (100:1 or 50:1, *v*/*v*)). A yellow oil; IR (film): ν = 3363, 3018, 2992, 1711, 1670, 1216, 1053, 755 cm^−1^; ^1^H-NMR (200 MHz, CDCl_3_): δ = 9.30 (d, *J* = 2.2 Hz, 1H, H_aromat._), 9.12 (d, *J* = 2.2 Hz, 1H, H_aromat._), 8.78 (dd, *J* = 7.3 Hz, *J* = 1.2 Hz, 1H, H_aromat._), 8.42 (dd, *J* = 8.3 Hz, *J* = 1.2 Hz, 1H, H_aromat._), 7.93 (dd, *J* = 8.3 Hz, *J* = 7.3 Hz, 1H, H_aromat._), 7.82 (s, 1H, *H*C5′), 5.52 (s, 2H, CH_2_), 4.48 (t, *J* = 5.3 Hz, 2H, PCH_2_CH_2_OCH_2_C*H*_2_), 4.12–3.98 (m, 4H, 2 × POC*H*_2_CH_3_), 3.78 (t, *J* = 5.3 Hz, 2H, PCCOC*H*_2_CH_2_), 3.66 (dt, *J* = 12.1 Hz, *J* = 7.6 Hz, 2H, PCH_2_C*H*_2_O), 2.05 (dt, *J* = 18.9 Hz, *J* = 7.6 Hz, 2H, PC*H*_2_CH_2_O), 1.29 (t, *J* = 7.1 Hz, 6H, 2 × POCH_2_C*H*_3_); ^13^C-NMR (151 MHz, CDCl_3_): δ = 162.76, 162.25, 146.40, 142.86, 135.67, 134.68, 131.06, 130.29, 129.10, 129.03, 124.65, 124.40, 124.37, 123.17, 68.96, 65.25 (PC*C*O), 61.68 (d, *J* = 6.0 Hz, PO*C*), 50.13, 35.57, 26.87 (d, *J* = 140.4 Hz, PC), 16.41 (d, *J* = 6.1 Hz, POC*C*); ^31^P-NMR (81 MHz, CDCl_3_): δ = 28.70 ppm. Anal. Calcd. for C_23_H_26_N_5_O_8_P: C, 51.98; H, 4.93; N, 13.18. Found: C, 51.70; H, 4.67; N, 13.15.

*Diethyl 2-{4-[(5-Nitro-1,3-dioxo-1H-benzo[de]isoquinolin-2(3H)-yl)methyl]-1H-1,2,3-triazol-1-yl}acetamido-methylphosphonate* (**16i**): Yield 91%. A white powder; m.p. 217–219 °C; IR (KBr): ν = 3287, 3075, 2986, 2854, 1709, 1687, 1229, 1031, 758 cm^−1^; ^1^H-NMR (200 MHz, DMSO-*d*_6_): δ = 9.51 (d, *J* = 2.2 Hz, 1H, H_aromat._), 9.00 (d, *J* = 2.2 Hz, 1H, H_aromat._), 8.82 (dd, *J* = 7.5 Hz, *J* = 0.6 Hz, 1H, H_aromat._), 8.72 (dd, *J* = 8.2 Hz, *J* = 0.6 Hz, 1H, H_aromat._), 8.70 (brt, *J* = 2.8 Hz, 1H, NH), 8.09 (dd, *J* = 8.2 Hz, *J* = 7.5 Hz, 1H, H_aromat._), 8.03 (s, 1H, *H*C5′), 5.36 (s, 2H, CH_2_), 5.10 (s, 2H), 4.19–3.97 (m, 4H, 2 × POC*H*_2_CH_3_), 3.59 (dd, *J* = 11.8 Hz, *J* = 6.0 Hz, 2H, PC*H*_2_NH), 1.19 (t, *J* = 7.1 Hz, 6H, 2 × POCH_2_C*H*_3_); ^13^C-NMR (151 MHz, DMSO-*d*_6_): δ = 165.93 (d, *J* = 5.2 Hz, C=O), 162.97, 162.51, 146.36, 142.85, 137.00, 134.60, 131.39, 130.37, 130.04, 129.76, 125.41, 124.35, 123.57, 122.91, 62.30 (d, *J* = 6.5 Hz, PO*C*), 51.87, 35.97, 34.65 (d, *J* = 155.5 Hz, PC), 16.65 (d, *J* = 5.6 Hz, POC*C*); ^31^P-NMR (81 MHz, DMSO-*d*_6_): δ = 22.27 ppm. Anal. Calcd. for C_22_H_23_N_6_O_8_P: C, 49.82; H, 4.37; N, 15.84. Found: C, 49.88; H, 4.07; N, 15.64.

*Diethyl {4-[(5-Amino-1,3-dioxo-1H-benzo[de]isoquinolin-2(3H)-yl)methyl]-1H-1,2,3-triazol-1-yl}methylphosphonate* (**17a**): Yield 75% (after column chromatography with chloroform–methanol mixtures (100:1 and 50:1, *v*/*v*)). A yellow oil; IR (film): ν = 3430, 3352, 3234, 2983, 2932, 1698, 1660, 1623, 1236, 1020, 784, 748 cm^−1^; ^1^H-NMR (600 MHz, DMSO-*d*_6_): δ = 8.10 (d, *J* = 7.2 Hz, 1H, H_aromat._), 8.07 (d, *J* = 8.2 Hz, 1H, H_aromat._), 8.00 (d, *J* = 2.2 Hz, 1H, H_aromat._), 7.95 ( s, 1H, *H*C5′), 7.64 (dd, *J* = 8.2 Hz, *J* = 7.2 Hz, 1H, H_aromat._), 7.32 (d, *J* = 2.2 Hz, 1H, H_aromat._), 6.01 (s, 2H, NH_2_), 5.30 (s, 2H, CH_2_), 5.01 (d, *J* = 12.9 Hz, 2H, PCH_2_), 4.05–3.98 (m, 4H, 2 × POC*H*_2_CH_3_), 1.19 (t, *J* = 7.0 Hz, 6H, 2 × POCH_2_C*H*_3_); ^13^C-NMR (151 MHz, DMSO-*d*_6_): δ = 164.00, 163.82, 148.41, 143.74, 134.10, 132.23, 127.46, 126.08, 124.85, 122.91, 122.35, 122.11, 121.11, 112.45, 63.02 (d, *J* = 6.3 Hz, POC), 45.10 (d, *J* = 150.3 Hz, PC), 35.51, 16.47 (d, *J* = 5.8 Hz, POC*C*); ^31^P-NMR (243 MHz, DMSO-*d*_6_): δ = 17.22 ppm. Anal. Calcd. for C_20_H_22_N_5_O_5_P: C, 54.18; H, 5.00; N, 15.80. Found: C, 54.36; H, 4.83; N, 15.84.

*Diethyl 2-{4-[(5-Amino-1,3-dioxo-1H-benzo[de]isoquinolin-2(3H)-yl)methyl]-1H-1,2,3-triazol-1-yl}ethylphosphonate* (**17b**): Yield 75% (after column chromatography with chloroform–methanol mixtures (100:1 and 50:1, *v*/*v*)). A yellow oil; IR (film): ν = 3443, 3356, 3233, 3147, 3063, 2986, 1698, 1661, 1626, 1220, 1027, 784, 752 cm^−1^; ^1^H-NMR (200 MHz, CDCl_3_): δ = 8.26 (dd, *J* = 7.2 Hz, *J* = 1.1 Hz, 1H, H_aromat._), 7.98 (d, *J* = 2.4 Hz, 1H, H_aromat._), 7.86 (dd, *J* = 8.3 Hz, *J* = 1.1 Hz, 1H, H_aromat._), 7.70 (s, 1H, *H*C5′), 7.54 (dd, *J* = 8.3 Hz, *J* = 7.2 Hz, 1H, H_aromat._), 7.22 (d, *J* = 2.4 Hz, 1H, H_aromat._), 5.46 (s, 2H, CH_2_), 4.62–4.49 (m, 2H, PCH_2_C*H*_2_), 4.30 (s, 2H, NH_2_), 4.12–3.97 (m, 4H, 2 × POC*H*_2_CH_3_), 2.47–2.30 (m, 2H, PC*H*_2_), 1.26 (t, *J* = 7.2 Hz, 6H, 2 × POCH_2_C*H*_3_); ^13^C-NMR (151 MHz, CDCl_3_): δ = 164.13, 163.84, 145.81, 143.92, 133.37, 131.85, 127.25, 126.92, 123.70, 123.02, 122.26, 122.19, 122.02, 113.90, 62.12 (d, *J* = 6.4 Hz, POC), 44.49 (PC*C*), 35.15, 27.23 (d, *J* = 141.4 Hz, PC), 16.32 (d, *J* = 5.7 Hz, POC*C*); ^31^P-NMR (81 MHz, CDCl_3_): δ = 26.33 ppm. Anal. Calcd. for C_21_H_24_N_5_O_5_P: C, 55.14; H, 5.29; N, 15.31. Found: C, 55.36; H, 5.06; N, 15.25.

*Diethyl 3-{4-[(5-Amino-1,3-dioxo-1H-benzo[de]isoquinolin-2(3H)-yl)methyl]-1H-1,2,3-triazol-1-yl}propylphosphonate* (**17c**): Yield 79% (after column chromatography with chloroform–methanol mixtures (100:1 and 50:1, *v*/*v*)). A white powder; m.p. 180–182 °C; IR (KBr): ν = 3451, 3343, 3235, 3148, 3067, 2986, 1700, 1652, 1619, 1222, 1029, 794, 759 cm^−1^; ^1^H-NMR (600 MHz, CDCl_3_): δ = 8.28 (dd, *J* = 7.3 Hz, *J* = 0.8 Hz, 1H, H_aromat._), 8.00 (d, *J* = 2.3 Hz, 1H, H_aromat._), 7.87 (dd, *J* = 8.2 Hz, *J* = 0.8 Hz, 1H, H_aromat._), 7.70 (s, 1H, *H*C5′), 7.56 (dd, *J* = 8.2 Hz, *J* = 7.3 Hz, 1H, H_aromat._), 7.23 (d, *J* = 2.3 Hz, 1H, H_aromat._), 5.49 (s, 2H, CH_2_), 4.42 (t, *J* = 6.9 Hz, 2H, PCH_2_CH_2_C*H*_2_), 4.33 (s, 2H, NH_2_), 4.11–4.04 (m, 4H, 2 × POC*H*_2_CH_3_), 2.21 (dqu, *J* = 18.6 Hz, *J* = 6.9 Hz, 2H, PCH_2_C*H*_2_), 1.73 (dt, *J* = 18.6 Hz, *J* = 7.7 Hz, 2H, PC*H*_2_), 1.30 (t, *J* = 7.1 Hz, 6H, 2 × POCH_2_C*H*_3_); ^13^C-NMR (151 MHz, CDCl_3_): δ = 164.01, 163.83, 148.37, 143.72, 134.07, 132.14, 127.43, 126.05, 123.71, 122.96, 122.33, 122.16, 122.15, 112.40, 61.52 (d, *J* = 6.7 Hz, POC), 49.77 (d, *J* = 15.2 Hz, PCC*C*), 35.68, 23.83 (d, *J* = 3.5 Hz, PC*C*), 22.32 (d, *J* = 143.2 Hz, PC), 16.65 (d, *J* = 5.5 Hz, POC*C*); ^31^P-NMR (243 MHz, CDCl_3_): δ = 30.01 ppm. Anal. Calcd. for C_22_H_26_N_5_O_5_P: C, 56.05; H, 5.56; N, 14.86. Found: C, 55.80; H, 5.41; N, 14.60.

*Diethyl 4-{4-[(5-Amino-1,3-dioxo-1H-benzo[de]isoquinolin-2(3H)-yl)methyl]-1H-1,2,3-triazol-1-yl}butylphosphonate* (**17d**): Yield 70% (after column chromatography with chloroform–methanol mixtures (100:1 and 50:1, *v*/*v*)). A yellow powder; m.p. 218–220 °C; IR (KBr): ν = 3451, 3343, 3235, 3148, 3067, 2986, 1700, 1652, 1619, 1222, 1029, 794, 759 cm^−1^; ^1^H-NMR (600 MHz, CDCl_3_): δ = 8.28 (dd, *J* = 7.3 Hz, *J* = 0.8 Hz, 1H, H_aromat._), 8.00 (d, *J* = 2.3 Hz, 1H, H_aromat._), 7.87 (dd, *J* = 8.2 Hz, *J* = 0.8 Hz, 1H, H_aromat._), 7.70 (s, 1H, *H*C5′), 7.56 (dd, *J* = 8.2 Hz, *J* = 7.3 Hz, 1H, H_aromat._), 7.23 (d, *J* = 2.3 Hz, 1H, H_aromat._), 5.49 (s, 2H, CH_2_), 4.42 (t, *J* = 6.9 Hz, 2H, PCCCC*H*_2_),4.33 (s, 2H, NH_2_), 4.20–4.00 (m, 4H, 2 × POC*H*_2_CH_3_), 2.02 (qu, *J* = 6.9 Hz, 2H, PCCC*H*_2_), 1.80–1.60 (m, 4H, PCC*H*_2_ and PC*H*_2_), 1.30 (t, *J* = 7.0 Hz, 6H, 2 × POCH_2_C*H*_3_); ^13^C-NMR (151 MHz, DMSO-*d*_6_): δ = 163.99, 163.81, 148.39, 143.61, 134.08, 132.15, 127.42, 126.04, 123.55, 122.97, 122.34, 122.17, 121.14, 112.38, 61.26 (d, *J* = 6.0 Hz, 2 × POC), 49.16, 35.67, 30.64 (d, *J* = 16.0 Hz, PCC*C*), 24.28 (d, *J* = 138.9 Hz, PC), 19.62 (d, *J* = 4.7 Hz, PC*C*), 16.69 (d, *J* = 5.7 Hz, POC*C*); ^31^P-NMR (243 MHz, CDCl_3_): δ = 30.91 ppm. Anal. Calcd. for C_23_H_28_N_5_O_5_P: C, 56.90; H, 5.81; N, 14.43. Found: C, 56.95; H, 5.75; N, 14.69.

*Diethyl 2-{4-[(5-Amino-1,3-dioxo-1H-benzo[de]isoquinolin-2(3H)-yl)methyl]-1H-1,2,3-triazol-1-yl}-1-hydroxyethylphosphonate* (**17e**): Yield 80% (after crystallization from a methanol–diethyl ether mixture). A white powder; m.p. 170–172 °C; IR (KBr): ν = 3420, 3330, 3233, 3154, 2982, 1700, 1650, 1620, 1228, 1049, 1016, 797, 757 cm^−1^; ^1^H-NMR (600 MHz, DMSO-*d*_6_): δ = 8.10 (d, *J* = 7.3 Hz, 1H, H_aromat._), 8.05 (d, *J* = 8.2 Hz, 1H, H_aromat._), 8.00 (s, 1H, *H*C5′), 7.99 (d, *J* = 2.3 Hz, 1H, H_aromat._), 7.63 (dd, *J* = 8.2 Hz, *J* = 7.3 Hz, 1H, H_aromat._), 7.30 (d, *J* = 2.3 Hz, 1H, H_aromat._), 6.00 (s, 2H, NH_2_), 5.28 (s, 2H, CH_2_), 4.52 (dt, *J* = 14.2 Hz, *J* = 3.7 Hz, 1H, PCC*H*_a_H_b_), 4.38 (ddd, *J* = 14.2 Hz, *J* = 10.1 Hz, *J* = 7.0 Hz, 1H, PCCH_a_*H*_b_), 4.23–4.18 (m, 1H, PCH), 4.07–4.00 (m, 4H, 2 × POC*H*_2_CH_3_), 1.21 (t, *J* = 7.0 Hz, 6H, 2 × POCH_2_C*H*_3_); ^13^C-NMR (151 MHz, DMSO-*d*_6_): δ = 163.35, 163.30, 143.25, 137.05, 134.17, 131.67, 129.82, 127.07, 126.51, 123.20, 122.98, 122.19, 122.04, 121.04, 120.83, 120.51, 67.57 (d, *J =* 168.4 Hz, P*C*), 63.36 (d, *J* = 6.8 Hz, POC), 63.24 (d, *J* = 6.8 Hz, POC), 52.46 (d, *J* = 11.7 Hz, PC*C*), 34.41, 16.60 (d, *J* = 4.7 Hz, 2 × POC*C*); ^31^P-NMR (243 MHz, DMSO-*d*_6_): δ = 25.85 ppm. Anal. Calcd. for C_21_H_24_N_5_O_6_P: C, 53.28; H, 5.11; N, 14.79. Found: C, 53.55; H, 5.35; N, 14.59.

*Diethyl 3-{4-[(5-Amino-1,3-dioxo-1H-benzo[de]isoquinolin-2(3H)-yl)methyl]-1H-1,2,3-triazol-1-yl}-2-hydroxypropylphosphonate* (**17f**): Yield 75%. (after crystallization from a methanol–diethyl ether mixture). An orange powder; m.p. 222–224 °C; IR (KBr): ν = 3437, 3333, 3234, 3140, 3068, 3034, 2922, 1653, 1616, 1223, 1049, 778, 744 cm^−1^; ^1^H-NMR (600 MHz, DMSO-*d*_6_): δ = 8.10 (d, *J* = 6.9 Hz, 1H, H_aromat._), 8.06 (d, *J* = 8.1 Hz, 1H, H_aromat._), 8.00 (d, *J* = 2.2 Hz, 1H, H_aromat._), 7.93 (s, 1H, *H*C5′), 7.63 (dd, *J* = 8.1 Hz, *J* = 6.9 Hz, 1H, H_aromat._), 7.31 (t, *J* = 2.2 Hz, 1H, H_aromat._), 6.00 (brs, 2H, NH_2_), 5.35 (brs, 1H, OH), 5.29 (s, 2H, CH_2_), 4.45 (dd, *J* = 13.8 Hz, *J* = 3.6 Hz, 1H, PCCC*H*_a_H_b_), 4.26 (dd, *J* = 13.8 Hz, *J* = 7.7 Hz, 1H, PCCCH_a_*H*_b_), 4.15–4.09 (m, 1H, PCCH), 4.02–3.93 (m, 4H, 2 × POC*H*_2_CH_3_), 1.97 (ddd, *J* = 18.0 Hz, *J* = 15.4 Hz, *J* = 5.3 Hz, 1H, PC*H*_a_H_b_), 1.88 (ddd, *J* = 18.0 Hz, *J* = 15.4 Hz, *J* = 7.1 Hz, 1H, PCH_a_*H*_b_), 1.21 (t, *J* = 7.0 Hz, 3H, POCH_2_C*H*_3_), 1.20 (t, *J* = 7.0 Hz, 3H, POCH_2_C*H*_3_); ^13^C-NMR (151 MHz, DMSO-*d*_6_): δ = 164.02, 163.84, 148.39, 143.29, 134.09, 132.16, 127.45, 126.05, 124.65, 122.98,122.34, 122.18, 121.14, 112.39, 65.45 (d, *J =* 1.8 Hz, PC*C*), 61.62 (d, *J* = 6.4 Hz, POC), 61.42 (d, *J* = 6.4 Hz, POC), 55.92 (d, *J* = 13.0 Hz, PCC*C*), 35.62, 31.47 (d, *J* = 137.0 Hz, PC), 16.66 (d, *J* = 5.9 Hz, 2 × POC*C*); ^31^P-NMR (243 MHz, DMSO-*d*_6_): δ = 27.89 ppm. Anal. Calcd. for C_22_H_26_N_5_O_6_P: C, 54.21; H, 5.38; N, 14.37. Found: C, 53.92; H, 5.29; N, 14.15.

*Diethyl 2-{4-[(5-Amino-1,3-dioxo-1H-benzo[de]isoquinolin-2(3H)-yl)methyl]-1H-1,2,3-triazol-1-yl}ethoxy-methylphosphonate* (**17g**): Yield 77% (after column chromatography with chloroform–methanol mixtures (100:1 or 50:1, *v*/*v*)). A yellow powder; m.p. 221–223 °C; IR (KBr): ν = 3438, 3339, 3233, 3145, 2980, 2890, 1701, 1651, 1620, 1222, 1048, 1024, 791, 743 cm^−1^; ^1^H-NMR (600 MHz, DMSO-*d*_6_): δ = 8.10 (d, *J* = 7.2 Hz, 1H, H_aromat._), 8.05 (d, *J* = 8.2 Hz, 1H, H_aromat._), 8.00 (d, *J* = 2.2 Hz, 1H, H_aromat._), 7.96 (s, 1H, *H*C5′), 7.63 (dd, *J* = 8.2 Hz, *J* = 7.2 Hz, 1H, H_aromat._), 7.31 (t, *J* = 2.2 Hz, 1H, H_aromat._), 6.00 (brs, 2H, NH_2_), 5.29 (s, 2H, CH_2_), 4.50 (t, *J* = 5.0 Hz, 2H, PCH_2_OCH_2_C*H*_2_), 3.92–3.88 (m, 6H, 2 × POC*H*_2_CH_3_, PCOC*H*_2_CH_2_), 3.80 (d, *J* = 8.3 Hz, 2H, PCH_2_O), 1.12 (t, *J* = 7.1 Hz, 6H, 2 × POCH_2_C*H*_3_); ^13^C-NMR (151 MHz, DMSO-*d*_6_): δ = 164.04, 163.80, 148.40, 143.56, 134.09, 132.17, 127.44, 126.06, 123.97, 122.97, 122.35, 122.17, 121.15, 112.40, 71.00 (d, *J* = 11.6 Hz, PCO*C*), 64.31 (d, *J* = 163.1 Hz, PC), 62.16 (d, *J* = 6.3 Hz, PO*C*), 49.47, 35.63, 16.63 (d, *J* = 5.3 Hz, 2 × POC*C*); ^31^P-NMR (243 MHz, DMSO-*d*_6_): δ = 20.69 ppm. Anal. Calcd. for C_22_H_26_N_5_O_6_P: C, 54.21; H, 5.38; N, 14.37. Found: C, 54.38; H, 5.46; N, 14.54.

*Diethyl 2-(2-{4-[(5-Amino-1,3-dioxo-1H-benzo[de]isoquinolin-2(3H)-yl)methyl]-1H-1,2,3-triazol-1-yl}ethoxy)ethylphosphonate* (**17h**): Yield 77% (after column chromatography with chloroform–methanol mixtures (100:1 or 50:1, *v*/*v*)). An orange powder; m.p. 180–183 °C; IR (KBr): ν = 3434, 3353, 3230, 2981, 2927, 1698, 1659, 1623, 1222, 1025, 787, 748 cm^−1^; ^1^H-NMR (600 MHz, CDCl_3_): δ = 8.33 (d, *J* = 7.2 Hz, 1H, H_aromat._), 8.06 (d, *J* = 2.2 Hz, 1H, H_aromat._), 7.82 (d, *J* = 8.2 Hz, 1H, H_aromat._), 7.78 (s, 1H, *H*C5′), 7.54 (dd, *J* = 8.2 Hz, *J* = 7.2 Hz,1H, H_aromat._), 7.18 (d, *J* = 2.2 Hz, 1H, H_aromat._), 6.00 (brs, 2H, NH_2_), 5.49 (s, 2H, CH_2_), 4.59 (t, *J* = 5.1 Hz, 2H, PCH_2_CH_2_OCH_2_C*H*_2_), 4.19–4.02 (m, 4H, 2 × POC*H*_2_CH_3_), 3.78 (t, *J* = 5.1 Hz, 2H, PCCOC*H*_2_CH_2_), 3.65 (dt, *J* = 15.4 Hz, *J* = 6.9 Hz, 2H, PCH_2_C*H*_2_O), 2.16 (dt, *J* = 18.6 Hz, *J* = 6.9 Hz, 2H, PC*H*_2_CH_2_O), 1.35 (t, *J* = 7.1 Hz, 6H, 2 × POCH_2_C*H*_3_); ^13^C-NMR (151 MHz, DMSO-*d*_6_): δ = 164.02, 163.84, 148.38, 143.56, 134.08, 132.13, 127.41, 126.03, 124.00, 123.00, 122.33, 122.20, 121.16, 112.37, 68.69, 64.73(PC*C*O), 61.36 (d, *J* = 6.4 Hz, PO*C*), 49.71, 35.68, 26.28 (d, *J* = 137.2 Hz, PC), 16.64 (d, *J* = 5.6 Hz, POC*C*); ^31^P-NMR (243 MHz, CDCl_3_): δ = 27.84 ppm. Anal. Calcd. for C_23_H_28_N_5_O_6_P: C, 55.09; H, 5.63; N, 13.97. Found: C, 55.12; H, 5.36; N, 13.70.

*Diethyl 2-{4-[(5-Amino-1,3-dioxo-1H-benzo[de]isoquinolin-2(3H)-yl)methyl]-1H-1,2,3-triazol-1-yl}acetamido-methylphosphonate* (**17i**): Yield 96%. A yellow powder; m.p. 222–224 °C; IR (KBr): ν = 3447, 3374, 3225, 3146, 2991, 2927, 1695, 1649, 1619, 1223, 1021, 779, 744 cm^−1^; ^1^H-NMR (600 MHz, DMSO-*d*_6_): δ = 8.73 (t, *J* = 5.5 Hz, 1H, NHCO), 8.10 (d, *J* = 7.2 Hz, 1H, H_aromat._), 8.05 (d, *J* = 8.1 Hz, 1H, H_aromat._), 8.00 (d, *J* = 2.2 Hz, 1H, H_aromat._), 7.95 (s, 1H, *H*C5′), 7.63 (dd, *J* = 8.1 Hz, *J* = 7.2 Hz, 1H, H_aromat._), 7.31 (t, *J* = 2.2 Hz, 1H, H_aromat._), 6.00 (brs, 2H, NH_2_), 5.30 (s, 2H, CH_2_), 5.09 (s, 2H), 4.02–3.97 (m, 4H, 2 × POC*H*_2_CH_3_), 3.60 (dd, *J* = 11.8 Hz, *J* = 5.9 Hz, 2H, PC*H*_2_NH), 1.19 (t, *J* = 6.9 Hz, 6H, 2 × POCH_2_C*H*_3_); ^13^C-NMR (151 MHz, DMSO-*d*_6_): δ = 165.97 (d, *J* = 4.5 Hz, C=O), 164.00, 163.82, 148.39, 143.40, 134.08, 132.19, 127.45, 126.08, 125.18, 122.93, 122.35, 122.13, 121.12, 112.43, 62.32 (d, *J* = 6.2 Hz, PO*C*), 51.83, 35.58, 34.62 (d, *J* = 155.2 Hz, PC), 16.64 (d, *J* = 5.7 Hz, POC*C*); ^31^P-NMR (121.5 MHz, DMSO-*d*_6_): δ = 22.29 ppm. Anal. Calcd. for C_22_H_25_N_6_O_6_P: C, 52.80; H, 5.04; N, 16.79. Found: C, 52.71; H, 4.86; N, 16.53.

### 3.6. Antiviral Activity Assays

The compounds were evaluated against the following viruses: herpes simplex virus type 1 (HSV-1) strain KOS, thymidine kinase-deficient (TK^−^) HSV-1 KOS strain resistant to ACV (ACV^r^), herpes simplex virus type 2 (HSV-2) strains Lyons and G, varicella-zoster virus (VZV) strain Oka, TK^−^ VZV strain 07−1, human cytomegalovirus (HCMV) strains AD-169 and Davis, vaccinia virus Lederle strain, respiratory syncytial virus (RSV) strain Long, vesicular stomatitis virus (VSV), Coxsackie B4, Parainfluenza 3, Influenza virus A (subtypes H1N1, H3N2), influenza virus B, Reovirus-1, Sindbis, Reovirus-1, Punta Toro, human immunodeficiency virus type 1 strain III_B_ and human immunodeficiency virus type 2 strain ROD. The antiviral, other than anti-HIV, assays were based on inhibition of virus-induced cytopathicity or plaque formation in human embryonic lung (HEL) fibroblasts, African green monkey cells (Vero), human epithelial cells (HeLa) or Madin-Darby canine kidney cells (MDCK). Confluent cell cultures in microtiter 96-well plates were inoculated with 100 CCID_50_ of virus (1 CCID_50_ being the virus dose to infect 50% of the cell cultures) or with 20 plaque forming units (PFU) (VZV) in the presence of varying concentrations of the test compounds. Viral cytopathicity or plaque formation was recorded as soon as it reached completion in the control virus-infected cell cultures that were not treated with the test compounds. Antiviral activity was expressed as the EC_50_ or compound concentration required to reduce virus-induced cytopathogenicity or viral plaque formation by 50%.

### 3.7. Cytostatic Activity Assays

All assays were performed in 96-well microtiter plates. To each well were added (5–7.5) × 10^4^ tumor cells and a given amount of the test compound. The cells were allowed to proliferate for 48 h (murine leukemia L1210 cells) or 72 h (human lymphocytic CEM and human cervix carcinoma HeLa cells) at 37 °C in a humidified CO_2_-controlled atmosphere. At the end of the incubation period, the cells were counted in a Coulter counter. The IC_50_ (50% inhibitory concentration) was defined as the concentration of the compound that inhibited cell proliferation by 50%.

## 4. Conclusions

A novel series of diethyl {4-[(5-substituted-1,3-dioxo-1*H*-benzo[*de*]isoquinolin-2(3*H*)-yl)-methyl]-1*H*-1,2,3-triazol-1-yl}alkylphosphonates has been synthesized in good to excellent yields via Cu(I)-catalyzed Hüisgen dipolar cycloaddition of *N*-propargyl naphthalimides **7/8** and **11/12** with the respective azidoalkylphosphonates **13a**–**i** under microwave irradiation.

The synthesized phosphonates **14a**–**i**–**17a**–**i** were evaluated against a variety of DNA and RNA viruses and several of them appeared slightly active against VZV (EC_50_ = 27.6–91.5 μM). Among them, the compound **16b**, which showed no potency toward the TK^+^ VZV strain, was found the most active against the TK^−^ VZV strain (EC_50_ = 27.59 μM), with EC_50_ values comparable to reference drugs. On the other hand, compound **16d** exhibited the highest activity against TK^+^ VZV (EC_50_ = 29.91 μM), athough lower than that of reference compounds.

Cytostatic properties of compounds **14a**–**i**–**17a**–**i** were studied on L1210, CEM, HeLa and HMEC-1 cell lines and most of them were only slightly cytostatic for HeLa (IC_50_ = 29–130 µM) and L1210 cells (IC_50_ = 14–142 µM). Among all tested compounds **14a**–**i**–**17a**–**i** derivatives substituted with a bromine atom at C6 (**15b** and **15d**) were the most active. Based on a preliminary SAR analysis it was established that the presence of the 1,2,3-triazole unit is essential for the cytostatic activity. Furthermore, compounds with longer linkers [(CH_2_)_3_, (CH_2_)_4_ and CH_2_CH_2_OCH_2_CH_2_)] showed the higher cytostatic potency than those having shorter fragments [CH(OH)CH_2_ and CH_2_NHC(O)CH_2_].

## Figures and Tables

**Table 1:** The antiviral activity and cytotoxicity against varicella-zoster virus (VZV) in HEL cell cultures.

Compound	Antiviral Activity EC_50_ (μM) ^a^		Cytotoxicity (μM)	
	TK^+^ VZV Strain	TK^−^ VZV Strain	Cell Morphology (MCC) ^b^	Cell Growth (CC_50_) ^c^
**11**	48.9	>100	100	n.d.
**14c**	>100	64.47	>100	n.d.
**14d**	86.39	56.66	>100	n.d.
**14e**	69.93	>100	>100	n.d.
**14h**	>100	74.63	>100	n.d.
**15b**	34.2	38.07	>100	n.d.
**15d**	40.44	37.54	100	n.d.
**15f**	86.39	>100	>100	n.d.
**15h**	37.14	70.83	>100	n.d.
**16a**	39.11	64.47	>100	n.d.
**16b**	>100	27.59	100	n.d.
**16c**	32.82	47.82	>100	n.d.
**16d**	29.91	53.85	>100	n.d.
**16e**	83.63	>100	>100	n.d.
**16f**	56.66	91.45	>100	n.d.
**16g**	50.17	>100	>100	n.d.
**16h**	40.9	64.47	>100	n.d.
**Amonafide**	5.36	7.82	20	1.15
	3.18	6.48	20	3.25
**Acyclovir**	1.51	47.51	>440	>440
	1.15	29.35	>440	>440
**Brivudin**	0.013	24.95	>300	>300
	0.0078	13.72	>300	>300

^a^ Effective concentration required to reduce virus plaque formation by 50%. Virus input was 100 plaque forming units (PFU); ^b^ Minimum cytotoxic concentration that causes a microscopically detectable alternation of cell morphology; ^c^ Cytotoxic concentration required to reduce cell growth by 50%, n.d.—not determined.

**Table 2:** The inhibitory effect of the tested compounds against the proliferation of murine leukemia (L1210), human T-lymphocyte (CEM) and human cervix carcinoma cells (HeLa).

Compound	IC_50_^a^ (µM)			
	L1210	CEM	HeLa	HMEC-1
**7**	>250	206 ± 42	48 ± 8	138 ± 38
**8**	≥250	≥250	>250	>250
**11**	177 ± 16	229 ± 18	109 ± 5	>250
**14a**	>250	187 ± 25	>250	>250
**14b**	>250	148 ± 59	204 ± 65	>250
**14c**	≥250	124 ± 19	172 ± 20	≥250
**14d**	156 ± 40	119 ± 3	150 ± 11	180 ± 54
**14e**	>250	>250	≥250	≥250
**14f**	>250	158 ± 4	168 ± 26	>250
**14g**	>250	168 ± 29	215 ± 49	≥250
**14h**	196 ± 32	171 ± 25	154 ± 22	222 ± 39
**14i**	>250	≥250	>250	>250
**15a**	28 ± 1	60 ± 27	88 ± 5	185 ± 40
**15b**	14 ± 1	56 ± 20	62 ± 20	152 ± 1
**15c**	20 ± 3	52 ± 34	62 ± 1	152 ± 0
**15d**	17 ± 3	22 ± 0	55 ± 2	92 ± 43
**15e**	42 ± 15	196 ± 60	>250	>250
**15f**	23 ± 3	60 ± 13	87 ± 21	152 ± 1
**15g**	24 ± 3	66 ± 20	84 ± 16	152 ± 1
**15h**	26 ± 2	29 ± 11	61 ± 15	151 ± 0
**15i**	31 ± 2	137 ± 72	>250	>250
**16a**	66 ± 7	69 ± 1	44 ± 19	154 ± 1
**16b**	>250	176 ± 37	54 ± 21	118 ± 71
**16c**	34 ± 9	55 ± 23	29 ± 17	136 ± 22
**16d**	70 ± 52	95 ± 4	81 ± 4	126 ± 37
**16e**	111 ± 39	127 ± 14	132 ± 2	>250
**16f**	68 ± 11	71 ± 21	42 ± 21	155 ± 6
**16g**	71 ± 1	79 ± 4	76 ± 8	148 ± 3
**16h**	64 ± 11	66 ± 0	56 ± 16	152 ± 1
**16i**	>250	>250	86 ± 6	>250
**17a**	177 ± 76	214 ± 5	101 ± 78	n.d
**17b**	>250	120 ± 7	69 ± 12	n.d
**17c**	>250	≥250	≥250	n.d
**17d**	>250	>250	116 ± 60	n.d
**17e**	>250	>250	72 ± 58	n.d
**17f**	>250	>250	130 ± 77	n.d
**17g**	>250	>250	97 ± 42	n.d
**17h**	142 ± 2	109 ± 3	66 ± 3	n.d
**17i**	>250	>250	112 ± 58	n.d
**Amonafide**	1.0 ± 0.4	0.54 ± 0.50	2.0 ± 1.2	0.31 ± 0.00

^a^ 50% Inhibitory concentration or compound concentration required to inhibit tumor cell proliferation by 50%, n.d.—not determined.
